# Sequence Analysis and Structure Prediction of SARS-CoV-2 Accessory Proteins 9b and ORF14: Evolutionary Analysis Indicates Close Relatedness to Bat Coronavirus

**DOI:** 10.1155/2020/7234961

**Published:** 2020-10-20

**Authors:** Chittaranjan Baruah, Papari Devi, Dhirendra K. Sharma

**Affiliations:** ^1^Bioinformatics Laboratory (DBT-Star College), P.G. Department of Zoology, Darrang College, Tezpur, 784 001 Assam, India; ^2^TCRP Foundation, 781006, Guwahati, India; ^3^School of Biological Sciences, University of Science and Technology, Meghalaya, Baridua-793101, India

## Abstract

Severe acute respiratory syndrome coronavirus 2 (SARS-CoV-2) has a single-stranded RNA genome that encodes 14 open reading frames (ORFs), eight of which encode accessory proteins that allow the virus to infect the host and promote virulence. The genome expresses around 29 structural and nonstructural protein products. The accessory proteins of SARS-CoV-2 are not essential for virus replication but do affect viral release, stability, and pathogenesis and finally contribute to virulence. This paper has attempted the structure prediction and functional analysis of two such accessory proteins, 9b and ORF14, in the absence of experimental structures. Sequence analysis, structure prediction, functional characterization, and evolutionary analysis based on the UniProtKB reviewed the amino acid sequences of SARS-CoV-2 9b (P0DTD2) and ORF14 (P0DTD3) proteins. Modeling has been presented with the introduction of hybrid comparative and *ab initio* modeling. QMEANDisCo 4.0.0 and ProQ3 for global and local (per residue) quality estimates verified the structures as high quality, which may be attributed to structure-based drug design targets. Tunnel analysis revealed the presence of 1-2 highly active tunneling sites, perhaps which will able to provide certain inputs for advanced structure-based drug design or to formulate potential vaccines in the absence of a complete experimental structure. The evolutionary analysis of both proteins of human SARS-CoV-2 indicates close relatedness to the bat coronavirus. The whole-genome phylogeny indicates that only the new bat coronavirus followed by pangolin coronaviruses has a close evolutionary relationship with the novel SARS-CoV-2.

## 1. Introduction

Severe acute respiratory syndrome coronavirus 2 (SARS-CoV-2) is a positive-sense, single-stranded RNA virus with a genome size of 29,903 nucleotides in length. The 5′ terminus of the SARS-CoV-2 genome encodes a polyprotein (pp1ab), which is further cleaved into 15 nonstructural proteins (nsp-1 to nsp-10 and nsp-12 to nsp-16), whereas the 3′ terminus encodes four structural proteins (spike, envelope, membrane, and nucleocapsid) and eight accessory proteins (3a, 3b, p6, 7a, 7b, 8b, 9b, and ORF14) [1, 2]. The virus is the causative agent of coronavirus disease 2019 (COVID-19) and is contagious through human-to-human transmission. Previously identified human CoVs that cause human disease include alphaCoVs hCoV-NL63 and hCoV-229E and the betaCoVs HCoV-OC43, HKU1, severe acute respiratory syndrome CoV (SARS-CoV), and Middle East respiratory syndrome CoV (MERS-CoV) [3]. Among the seven strains coronaviruses (CoVs) discovered so far, three strains proved to be highly pathogenic (SARS-CoV, MERS-CoV, and 2019-nCoV), which caused endemic to severe CoV disease [4, 5]. The viruses can be classified into four genera: alpha, beta, gamma, and deltaCoVs [6]. The SARS-CoV and MERS-CoV infections can result in life-threatening diseases and have pandemic potential. SARS-CoV-2 is responsible for infection with special reference to the involvement of both the lower and upper respiratory tract [5, 7]. Furthermore, the potential for close contact between bats, civets, and humans in the wildlife trade in southern China, coupled with a possible propensity of these bats to foster CoV host-shifts, could explain SARS-like CoVs as the source of SARS-CoV [8].

To accommodate the wide spectrum of clinical presentations and outcomes of infections caused by SARS-CoV-2 [9], the WHO recently introduced the name COVID-19 (World Health Organization, 2020) to denote this disease. The acronym COVID-19 stands for “CO - corona,” “VI – viruses,” “D - disease,” and “19 - the year 2019” [10]. Despite the fact that COVID-19 has a death rate of 3.27% as of September, 27,236,916 confirmed cases with 891,031 confirmed deaths in a few months (December 8, 2019, to September 08, 2020) across 216 countries or territories are terrifying. Indeed, this virus is highly contagious, and the number of infected people can be doubled in less than seven days with a basic reproductive number (R0) of 2.2–2.7 [11]. In humans, SARS-CoV and SARS-CoV-2 are rapidly spread by respiratory droplets, airborne routes, or direct contact [12].

The viral genome encodes 29 proteins (*Nature* doi:10.1038/s41433-020-0790-7). The functions of a large number of SARS-CoV-2 ORFs are poorly understood or unknown. The accessory proteins are unique to SARS-CoV, as they have little homology in amino acid sequences with accessory proteins of other coronaviruses [13]. An accessory ORF14 was first described in SARS-CoV by Marra et al. [14, 15]. Understanding the complete proteome of SARS-CoV-2, including the accessory proteins, is the need of the hour for the final destination of drug/medicine. Although the complete genome of SARS-CoV-2 has been made available in the public domain databases, it has been observed in our previous study on SARS-CoV-2 proteome analysis [2] that the two “accessory” ORFs ORF13 (9b) and ORF14 are poorly studied in SARS-CoV-2, as both are not annotated in most of the completed genome sequences [2]. Given the similarity of SARS-CoV-2 to bat SARS-CoV-like coronaviruses, it is likely that bats serve as reservoir hosts for its progenitor. The SARS-CoV-2 spike protein optimized for binding to human-like ACE2 is the result of natural selection [16]. Therefore, the present study reports the *in silico* sequence analysis, structure prediction, and evolutionary analysis of two such accessory proteins, 9b and ORF14, of the newly emerged SARS-CoV-2.

## 2. Materials and Methods

### 2.1. Acquisition and Analysis of Sequences

**Figure 1:**
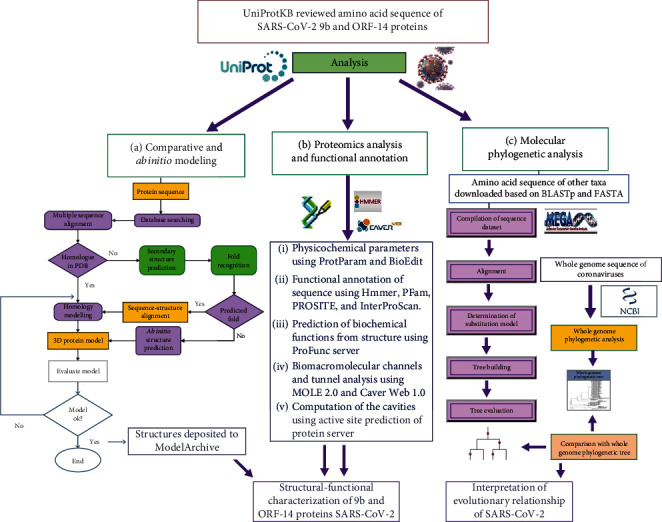
A conceptual framework of the present study for analysis of SARS-CoV-2 accessory proteins ORF9b and ORF14.

UniProtKB reviewed the amino acid sequences of SARS-CoV-2 9b (accession no. P0DTD2), and ORF14 protein (accession no. P0DTD3) was used in the present study. A conceptual framework of the workflow in the current study is represented in Figure 1. The amino acid sequences for different taxa were downloaded from UniProtKB for phylogenetic analysis based on BLASTp [17] and FASTA hits [18]. Data mining and sequence analyses were carried out using ExPASy proteomic tools (https://www.expasy.org/tools). The physicochemical parameters were computed using ProtParam [19] and BioEdit [20].

Genome sequences (16 nos.) of different coronavirus genomes with NCBI-IDs AY572034, AY572035, KF569996, MH734115, MG92481, MG923467, MT040335, MT040333, MT072864, MN996532, MT791905, MT451886, MT973427, DQ412042, DQ648856, and AY321118 were retrieved from NCBI genome database for construction of whole-genome phylogeny.

### 2.2. Comparative and Ab Initio Modeling

BlastP and FASTA searches were performed independently with PDB to know the existing structure from the PDB, for a suitable template for comparative modeling and to decide *ab initio* modeling requirements (Table 1). The significance of the BLAST results was assessed the e-value generated by the BLAST family of search algorithm and query coverage. The comparative modeling was carried out in the Modeller9.24 program [21], and *ab initio* modeling was done in Baker Rosetta Server (https://robetta.bakerlab.org/). The loop regions were modeled using the ModLoop server [22]. The final 3D structures with complete coordinates were obtained by optimization of the molecular probability density function of the Modeller 9.24 [23]. The computational protein structures were verified by using global and local (per residue) quality estimates of ProQ3 and QMEANDisCo 4.0.0 [24]. All the graphic presentations of the 3D structures were prepared using Chimera version 1.8.1 [25] and pyMOL 0.97rc [26].

### 2.3. Proteomics Analysis and Functional Annotation

Sequence-based functional annotation was carried out using Pfam (pfam.sanger.ac.uk/-), Hmmer version 3.3 [27], PFam, PROSITE, and InterProScan. ProFunc server [28] was used to identify the likely biochemical function of proteins from the predicted 3D structures. MOLE 2.0 [29] and Caver Web 1.0 [30] were used for the advanced analysis of biomacromolecular channels. The tunnel bottleneck radius and lengths were calculated in *Ångström* (Å) and throughput (estimated tunnel importance) calculated as *e*^−cost^, where *e* is Euler's number. Active site prediction of protein server [31] has been used for the computation of cavities in the target proteins.

### 2.4. Molecular Phylogenetic Analysis

The amino sequences used for phylogenetic analysis were aligned using ClustalW 1.6 [32] integrated in the MEGA X software [33]. The evolutionary history was inferred using maximum likelihood methods [34]. The percentage of replicate trees in which the associated taxa clustered together in the bootstrap test (1000 replicates) [35]. The initial tree(s) for the heuristic search were obtained automatically by applying the Neighbor-Join and BioNJ algorithms to a matrix of pairwise distances estimated using the JTT model and then selecting the topology with a superior log likelihood value. To verify the reliability of protein phylogeny (9b and ORF proteins), a whole-genome ML phylogenetic tree was constructed using the General Time Reversible model with Gamma distribution (G).

## 3. Results and Discussion

### 3.1. Tertiary Structures of 9b and ORF14

**Figure 2:**
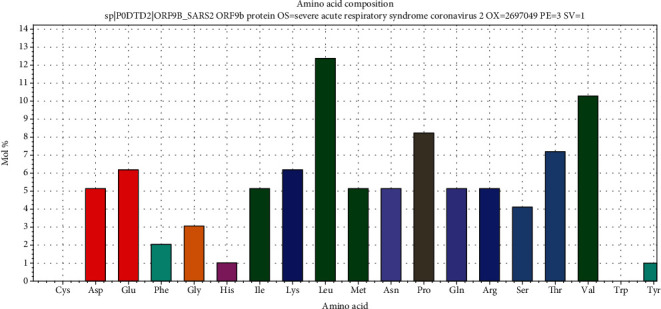
Amino acid composition of SARS-CoV-2 9b protein (leucine and valine-rich).

**Figure 3:**
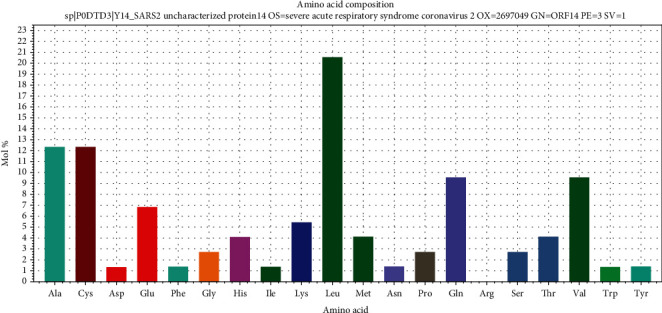
Amino acid composition of SARS-CoV-2 ORF14 protein (leucine and valine-rich).

**Figure 4:**
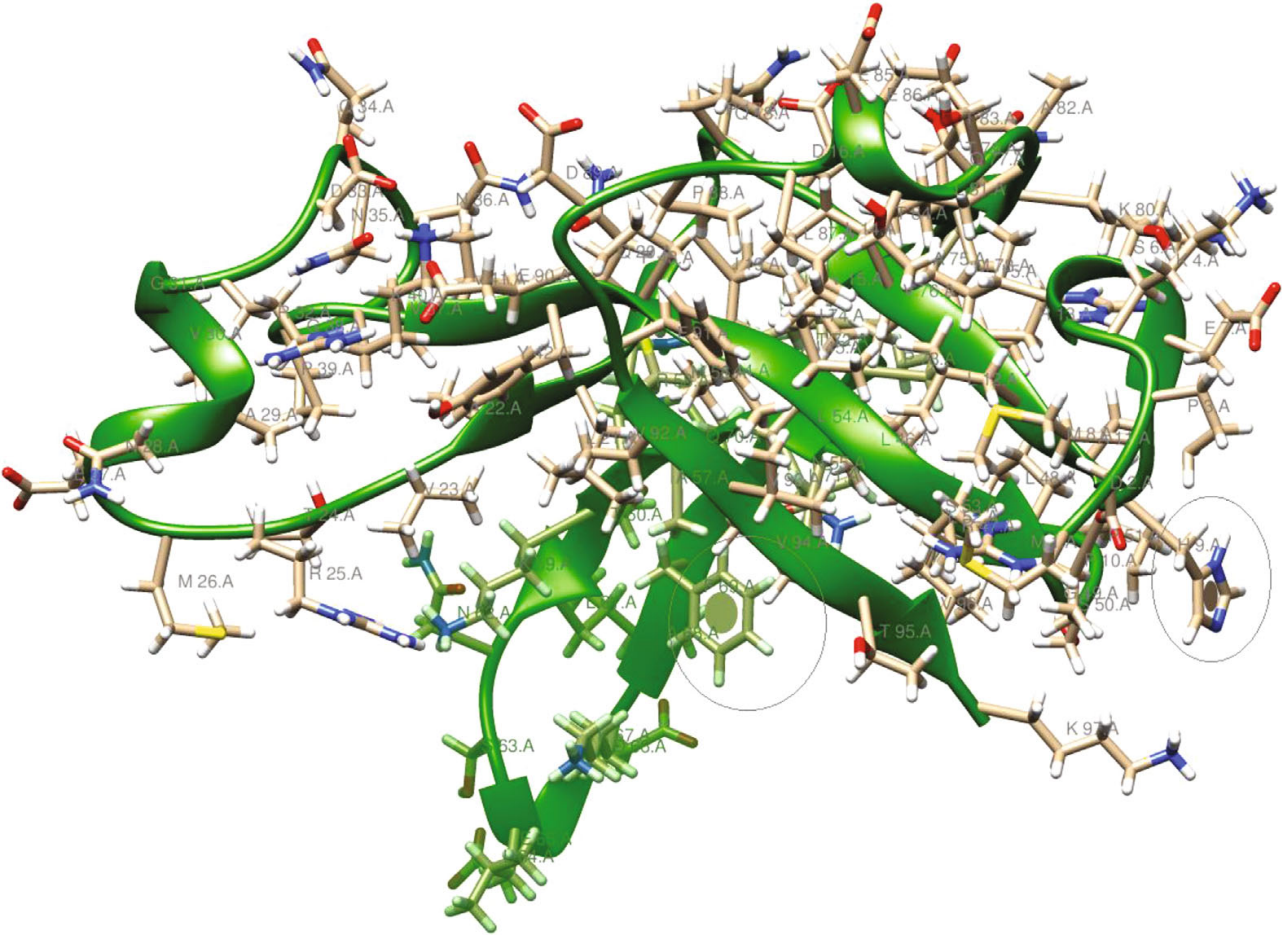
Structure of SARS-CoV-2 9b protein along with major active sites.

**Figure 5:**
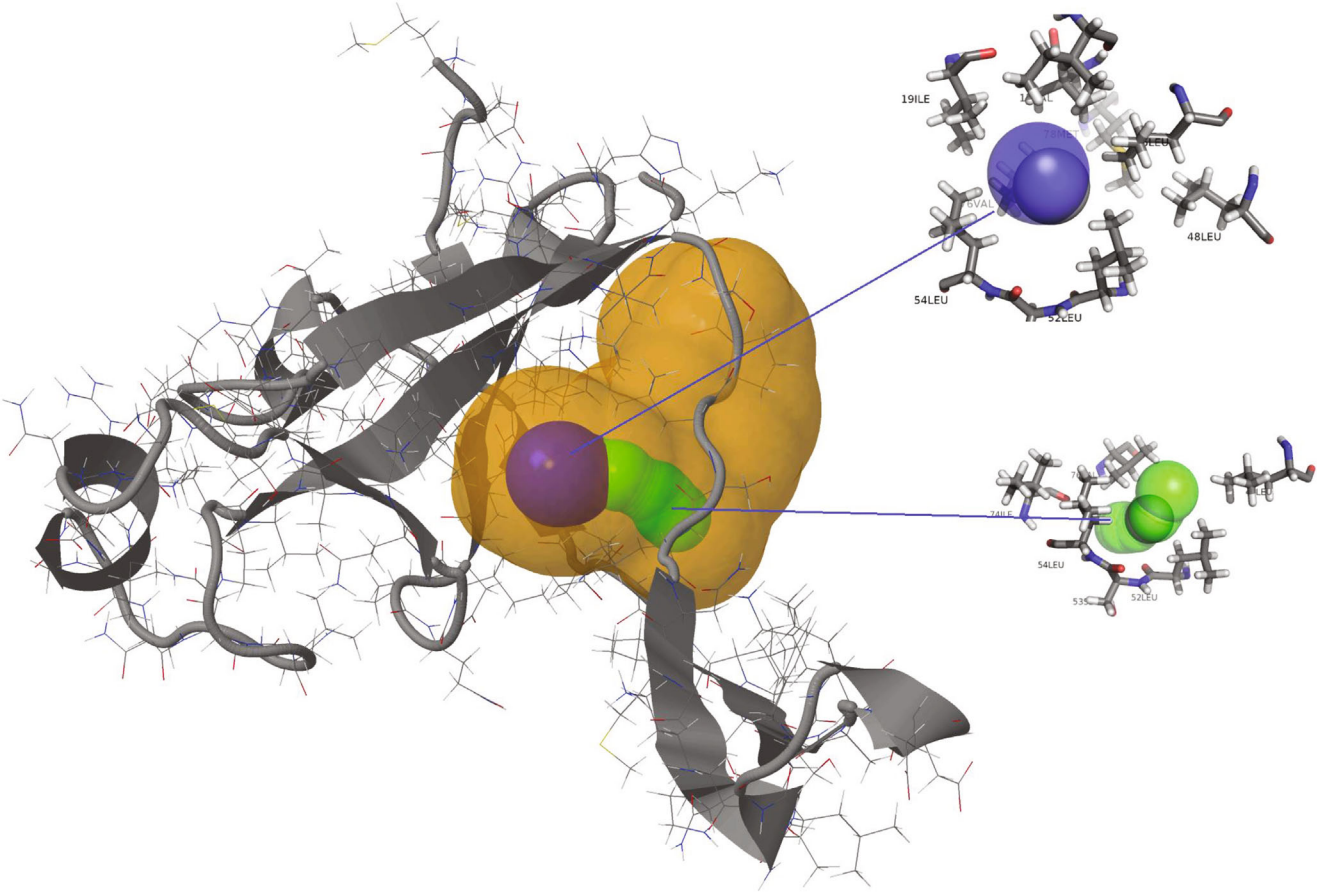
Structure of SARS-CoV-2 9b protein and its two high-throughput tunnels. Tunnels are colored on the basis of preferences in throughput values, i.e., tunnel-1 (blue) and tunnel-2 (green). The high relevance pocket is shown (yellow).

**Figure 6:**
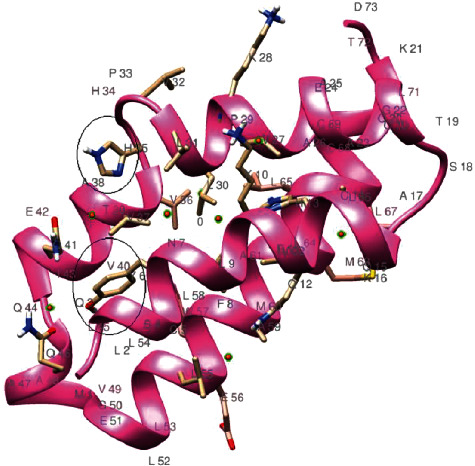
Structure of SARS-CoV-2 ORF14 protein along with major active sites.

**Figure 7:**
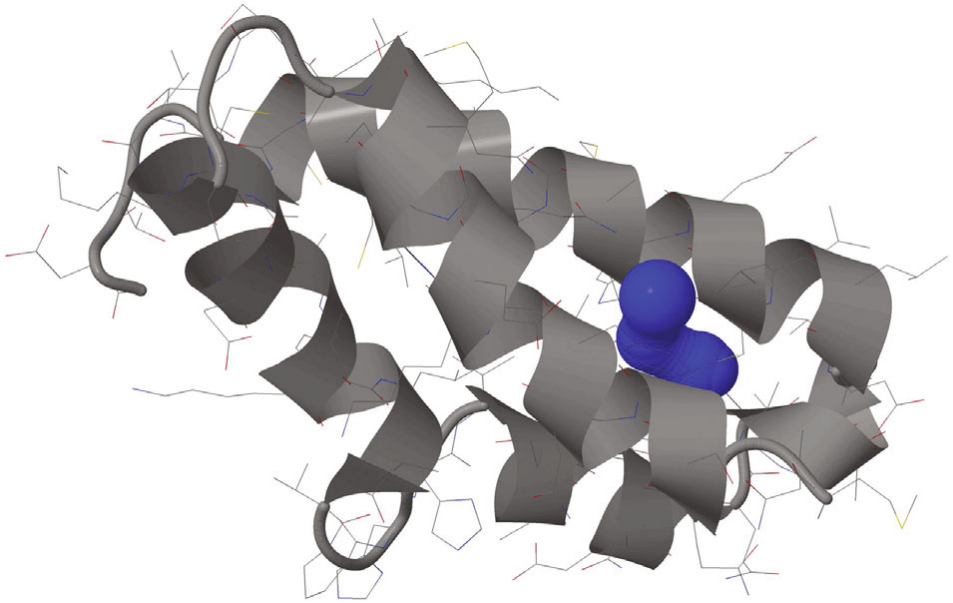
Structure of SARS-CoV-2 ORF14 protein along with its throughput tunnels (blue).

The 9b protein (P0DTD2) with molecular weight = 10796.10 Daltons is rich in leucine (12.37%) and valine (10.31%) (Figure 2). The ORF14 (P0DTD3) molecular weight = 8049.27 Daltons is rich in leucine (20.55%) and alanine (12.33%) (Figure 3). ProMotif analysis of the final predicted structure of 9b, modeled using comparative modeling, calculated 2 sheets, 2 beta hairpins, 7 strands, 4 helices, 9 beta turns, and 1 gamma turn (Table 1; Figures 4 and 5; Figure S1). ProMotif analysis of the predicted *ab initio* structure of ORF14 calculated 5 helices, 9 helix-helix interacts, and 2 beta turns (Table 1; Figures 6 and 7; Figure S2). The verified structures of the ORF9b and ORF14 proteins had qmean4 *z* scores of -1.64 (Global Score : 0.67 ± 0.09) and -1.18 (Global Score : 0.52 ± 0.11), respectively (Annexures 1 & 2). Precheck verification showed 94.0% and 95.5% residues in the most favored regions (A, B, L) of Ramachandran plot in 9b and ORF14 proteins, respectively. Structural verification in ERRAT revealed good quality of the models with quality factors of 97.56 and 100 for ORF9b and ORF14 proteins, respectively. The verification reports indicate the high reliability of the theoretical structures (Figures S3-S6).

The estimated high throughput tunnel-1 (blue) in the 9b protein is a bottleneck radius of 1.9 Å, length of 1.5 Å, distance to the surface of 1.5 Å, curvature of 1.0, throughput of 0.92, and number of residues 11; tunnel-2 (green) is a bottleneck radius of 1.5 Å, length of 5.4 Å, distance to surface of 4.9 Å, curvature of 1.1, throughput of 0.78, and number of residues 14 (Figure 5, Figures S7-S8). The estimated high throughput tunnel-1 (blue) in ORF14 protein is a bottleneck radius of 1.2 Å, length of 5.8 Å, distance to the surface of 4.9 Å, curvature of 1.2, throughput of 0.69, and number of residues 14 (Figure 7; Figure S9).

Structural comparison of SARS-CoV-2 9b protein (97 aa residues) with the crystal structure of SARS-CoV ORF9b protein (79 aa residues; PDB ID 2 CME), which shared 70.93% sequence identity (89% query coverage) showed minor differences in the number of strands, helices, and beta turns (SARS-CoV: 6 strands, 1 helix, 7 beta turns, 3 gamma turns; SARS-CoV-2: 7strands, 4 helices, 5 beta turns). This difference in the increase in the number of helices and strands may be due to increased sequence length in the SARS-CoV-2 9b protein. Changes in the DNA sequence will therefore affect both the conventional and alternative ORF, limiting the rate and extent to which the corresponding proteins can evolve [14]. The structure of SARS-CoV ORF9b is a 2-fold symmetric dimer constructed from two adjacent twisted *β* sheets [36]. Each of these sheets is formed from *β* strands contributed by both monomers, which form a highly interlocked architecture reminiscent of a handshake. The interdigitated nature of the ORF9b dimer rests on a highly unusual topology of largely antiparallel *β* sheets in which monomers wrap around each other [36].

The accessory ORF14 described only by Marra et al. [14] is still an uncharacterized protein, and very little is known about its structure and interactions. ORF14 has no significant sequence homology to proteins in other coronaviruses. It belongs to the group of proteins, named as predicted unknown proteins (PUPs), and is unique to SARS-CoV [15, 37]. Interactions among SARS-CoV accessory proteins were studied using a bimolecular fluorescence complementation assay [15, 37]. Self-interactions were observed with 9b and ORF14, indicating the formation of dimeric or multimeric complexes in the nucleus, similar to the findings of von Brunn et al. [15]. ORF9b and ORF14 interacted with themselves, indicating the formation of dimeric or multimeric complexes [15]. ORF9b and ORF14 self-interactions were also found in the co-Immunoprecipitation (CoIP) assay. *α*-Galactosidase and *β*-galactosidase assays of protein interactions of 9b-9b, 8a-9b, and ORF14-ORF14 demonstrated self-interactions [15, 37].

### 3.2. Proteomics Profiles of 9b and ORF14

**Figure 8:**
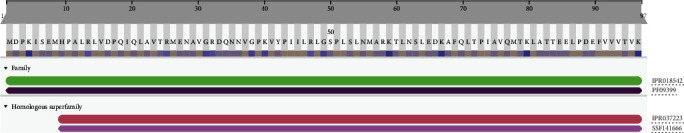
Protein family membership of SARS-CoV-2 protein 9b resembles the SARS-like protein (IPR018542) and the 9b SARS InterPro homologous superfamily (9-97; IPR037223).

The InterProScan Search Result of ORF9b protein has revealed that it belongs to protein family—protein 9b and SARS-like (IPR018542) (Figure 8). This is a family of proteins found in SARS and SARS-like coronaviruses. It includes protein 9b from SARS coronavirus 2 (SARS-CoV-2), human SARS coronavirus (SARS-CoV), and bat coronaviruses. Protein 9b is one of 8 accessory proteins in SARS-CoV [38]. The gene (ORF9b, also known as ORF13) that encodes this protein is included within the nucleocapsid (N) gene (alternative ORF) [39]. The ORF9b accessory protein is associated with the spike and nucleocapsid proteins and has unusual membrane-binding properties [14, 36]. SARS-CoV ORF9b has been shown to localize to the outer mitochondrial membrane and target mitochondrial antiviral signaling proteins (MAVS), suppressing innate immunity [40, 41]. Antibodies against SARS-CoV ORF9b have been found in patients, demonstrating that it is produced during infection [36, 42]. Protein 9b from SARS-CoV comprises 98 amino acids, the structure of which has a novel fold that forms a dimeric tent-like beta structure with an amphipathic surface, and a central hydrophobic cavity that binds lipid molecules [36]. This cavity is likely involved in membrane attachment [36]. The sequence of ORF9b is well conserved in different SARS isolates; however, there is little homology between protein 9b from SARS-CoV and the I-protein (protein 9b homologue) present in other coronaviruses [39, 43].

**Figure 9:**
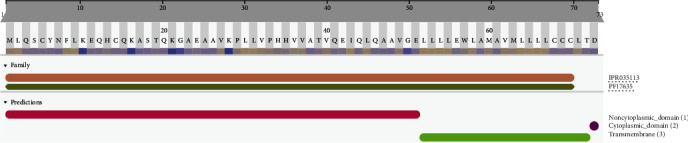
Protein family membership of SARS-CoV-2 protein 14 resembles SARS-like protein (IPR035113). The protein is with three domains: (i) noncytoplasmic domain (1-51), (ii) transmembrane region (52-72), and (iii) cytoplasmic domain (73-73).

InterProScan Search Result of ORF14 protein revealed its family of membership as protein 14, SARS-like (IPR035113) (*Protein 14_ SARS-like*) (Figure 9). This is a family of unknown functions found in SARS and SARS-like coronaviruses. It includes uncharacterized protein 14 from SARS coronavirus 2 (SARS-CoV-2), human SARS coronavirus (SARS-CoV), and bat coronavirus Rp3/2004 (SARS-like coronavirus Rp3) [14]. In SARS-CoV, ORF14 is completely contained within the ORF encoding the nucleocapsid protein (N) [38]. In SARS-CoV-2, uncharacterized protein 14 was predicted to contain one transmembrane helix. The ORF14 protein is with three domains: (i) noncytoplasmic domain (1-51), (ii) transmembrane region (52-72), and (iii) cytoplasmic domain (73-73).

Protein 9b shows its subcellular location as a host cytoplasmic vesicle membrane, peripheral membrane protein, and host cytoplasm that binds noncovalently to intracellular lipid bilayers. Gene ontology revealed the cellular components of the host cell cytoplasmic vesicle membrane, and the subunit structure is homodimer with binary interactions. ORF14 protein may play a role in host-virus interaction-subcellular location: membrane sequence analysis and single-pass membrane protein sequence analysis. The topology of gene ontology exhibits cellular components, integral components of the membrane, transmembrane, and transmembrane helices.

### 3.3. Functional Annotation of 9b and ORF14

PROSITE analysis of the 9b protein revealed three sites: (i) PS00006 CK2_PHOSPHO_SITE Casein kinase II phosphorylation site (24-27; 63-66; 83-86), (ii) PS00008 MYRISTYL N-myristoylation site (49--54), and (iii) PS00005 PKC_PHOSPHO_SITE Protein kinase C phosphorylation site (95-97).

The domain profile of ORF9b resembles the Sarbecovirus 9b domain profile (PROSITE entry PS51920). Coronaviruses are divided into four genera: *α*-coronavirus, *β*-coronavirus, *γ*-coronavirus, and delta-coronavirus. SARS, SARS-CoV-2, BatCoV RaTG13, and Bat-SARS-like coronavirus (BAT-SL-CoVZXC21 and BAT-SL-CoVZC45) belong to the Sarbecovirus subgenus of *β*-coronavirus.

Coronaviruses code for the characteristic proteins replicase polyprotein (pp1ab), spike (S), membrane (M), envelope (E), and nucleocapsid (N) proteins. In addition, Sarbecoviruses code for subgroup-specific accessory proteins that are thought to be dispensable for viral replication in cell culture but may be important for virus-host interactions and thus contribute to virus fitness.

To achieve the optimum output from their limited genomes, viruses frequently make use of alternative open reading frames, in which translation is initiated from a start codon within an existing gene and, being out of frame, gives rise to a distinct protein product. ORF9b codes for a small accessory protein of 98 amino acid residues, which are found in Sarbecovirus-infected cells. The ORF9b protein (p9b) has been shown to self-interact and interact with nsp5, nsp14, and the accessory protein p6. The function of p9b is unknown, although it has been suggested that it specifically recognizes and binds to intracellular vesicular. The 9b protein could have a role in membrane interactions during the assembly of the virus membranes [36, 44, 45].

The 9b domain has a fold with seven *β*-strands (PDB ID: 2CME). The *β*-strands from two molecules form two adjacent twisted *β*-sheets, resulting in a highly interlocked handshake structure that contains a hydrophobic central cavity, which binds to lipids and stabilizes the molecule (Meier et al., 2006) [36]. Protein 9b is a homodimer that plays a role in membrane interactions during the assembly of the virus.

PROSITE analysis of ORF14 protein also estimated three sites: (i)PS00005 PKC_PHOSPHO_SITE Protein kinase C phosphorylation site (aa 19-21), (ii) PS00008 MYRISTYL N-myristoylation site (aa 22-27), and (iii) PS00006 CK2_PHOSPHO_SITE Casein kinase II phosphorylation site (aa 39-42).

Casein kinase II (CK-2) is a protein serine/threonine kinase whose activity is independent of cyclic nucleotides and calcium. CK-2 phosphorylates many different proteins [46]. N-myristoylation site, an appreciable number of eukaryotic proteins are acylated by the covalent addition of myristate (a C14-saturated fatty acid) to their N-terminal residue via an amide linkage [47, 48]. The sequence specificity of the enzyme responsible for this modification, myristoyl CoA:protein N-myristoyl transferase (NMT), has been derived from the sequence of known N-myristoylated proteins and from studies using synthetic peptides [48]. In vivo, protein kinase C exhibits a preference for the phosphorylation of serine or threonine residues found close to a C-terminal basic residue (Meier et al., 2006; Liu *et al*., 2014). The presence of additional basic residues at the N- or C-terminal of the target amino acid enhances the *V*_max_ and *K*_m_ of the phosphorylation reaction [49].

The instability index values of the ORF9b and ORF14 proteins of SARS-COV-2 were 33.11 and 32.56, respectively, which classifies both proteins were stable (Table 2). The aliphatic indices of SARS-COV2 9b and ORF14 were 105.46 and 125.62, respectively, indicating high thermal stability in both proteins (Table 2). The grand average of hydropathicity (GRAVY) values of the 9b and ORF14 proteins are computed as -0.085 and 0.603, respectively, which indicates that protein 9b is hydrophilic and ORF14 is hydrophobic in nature (Table 2; Figures S10 and S11).

A comparison made in this study on the physicochemical parameters of ORF9b and ORF14 proteins among the different coronaviruses showed that ORF9 protein of SARS-CoV-2 has 76.53% sequence identity with *Rhinolophus affinis* coronavirus, 74.23% with human CoV, and 73.20% with bat SARS-CoV. The ORF14 protein of SARS-CoV-2 has 92.86% identity with the ORF14 protein of bat coronavirus, 78.57% with human SARS-CoV, 77.14% with civet and *Rhinolophus affinis* coronavirus. The ORF9 protein showed a wide range of isoelectric points from 4.9 (human and civet SARS-CoV) to 6.56 (human SARS-CoV-2). The instability index values of ORF9b of coronaviruses ranged from 33.11 (human SARS-CoV-2) to 41.80 (bat CoV) (Table 2). The instability index values of ORF14 of coronaviruses ranged from 25.59 (bat CoV) to 32.81 (*R. affinis* CoV) (Table 2). This indicates higher stability of the ORF14 protein than the ORF9 protein. The grand average of hydropathicity (GRAVY) values were computed in the range of -0.176 to -0.012 in ORF9b protein and 0.196 to 0.603 in ORF14, indicating that ORF9b protein is hydrophilic and ORF14 is hydrophobic in nature (Table 2). The physicochemical parameters, including amino acid composition, pI, instability index and hydropathicity of SARS-CoV-2, showed higher identity with bat SARS-CoV (Table 2).

The functional analysis results of Profunc have been presented in Table 3. Of the nine (09) estimated cavity points in the structure of the 9b protein, the cavity-1 produced by amino acids “NPQVDKGEYTAMIFRLS” is with xyz coordinates of 10.606, -3.416, and -5.832 and a volume of 1261 Ångström cube; the cavity-2 produced by the amino acids “DKQPRVELNTFYIAM” is with cavity point 10.014, -0.066, and 5.372 and a volume of 971 Ångström cube (Figure 5; Table S1). Out of the 10 potential cavities for computed active sites for the function of ORF14 protein, cavity-1 is represented by amino acids “HEPIATVLKWCDMY,” with xyz coordinates of -7.341, 17.615, and -5.427 and a volume of 662 Ångström cube; the cavity-2 with amino acids “PATIHQVLWKYENMCSF” is with a cavity point of -1.634, 12.400, and 3.280 and a volume of 494 Ångström cube (Figure 7; Table S2). ORF9b is an unusual membrane-binding protein with a long hydrophobic lipid-binding tunnel.

### 3.4. Molecular Phylogeny of the 9b and ORF14 Proteins

**Figure 10:**
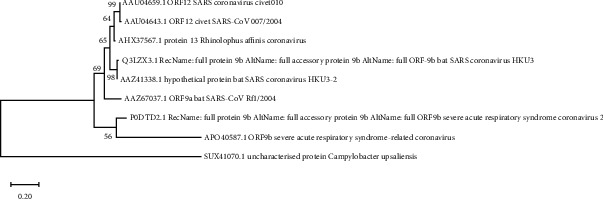
Evolutionary analysis of 9b protein of SARS-CoV-2 by Maximum Likelihood method and JTT matrix-based model (Jones and Taylor, 1992). The tree with the highest log likelihood (-1113.75) is shown. This analysis involved 9 amino acid sequences. There were a total of 141 positions in the final dataset. Evolutionary analyses were conducted in MEGA X.

Evolutionary analysis of the 9b and ORF14 proteins of SARS-CoV-2 was based on the Maximum Likelihood (ML) method and the JTT matrix-based model. The percentage of trees in which the associated taxa clustered together is shown next to the branches. The ML phylogenetic tree, based on the amino acid sequence of the 9b protein, revealed that it has close evolutionary relatedness with human SARS coronavirus (UniProtKB accession number APO40587) followed by the bat SARS-CoV (UniProtKB accession numbers AAZ67037, AAZ41338, and Q3LZX3 (Figure 10)).

**Figure 11:**
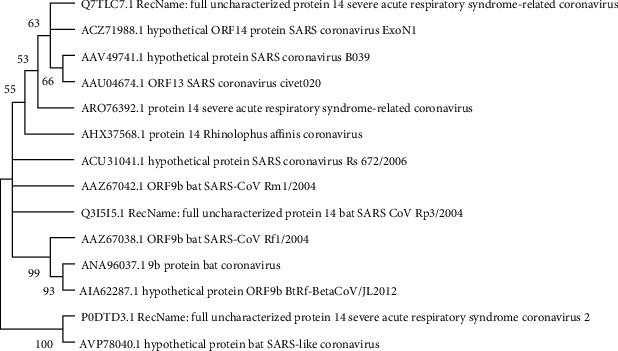
Evolutionary analysis of ORF14 protein of SARS-CoV-2 by Maximum Likelihood method and JTT matrix-based model (Jones and Taylor, 1992). The tree with the highest log likelihood (-497.56) is shown. The percentage of trees, in which the associated taxa clustered together, is shown next to the branches. This analysis involved 14 amino acid sequences. There were a total of 73 positions in the final dataset. Evolutionary analyses were conducted in MEGA X.

However, the ML phylogenetic tree based on the amino acid sequence of the human SARS-CoV2 ORF14 protein showed the closest evolutionary relationship with bat SARS-like coronaviruses (accession number AVP78040) with 100% boot strap support (Figure 11).

**Figure 12:**
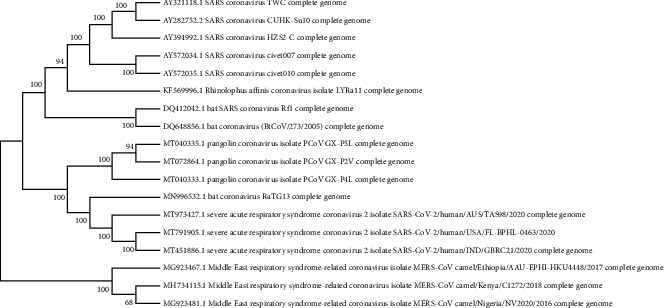
Evolutionary relationships of different coronaviruses based on whole-genome bootstrap phylogenetic analysis (ML tree).

The whole-genome phylogenetic tree strongly supports the protein phylogeny based on ORF9b and ORF14 proteins, indicating that the close evolutionary SARS-CoV-2 has very closely evolutionarily related to newly sequenced bat coronavirus RaTG13 genome/March 2020 from China (MN996532) followed by the pangolin coronavirus genome (MT040333, MT040335, MT072864) (Figure 12). Bat SARS-CoV Rf1/2004 (DQ412042) and bat CoV 273/2005 (DQ648856) along with human SARS-CoV and horseshoe *bat* (*Rhinolophus affinis*) formed a different clade in the whole-genome phylogeny, indicating rapid evolution of coronavirus. Moreover, all the pangolin coronavirus genomes sequenced in April 2020 (MT04033, MT072864, MT040335) were found to be sister taxa in the whole-genome phylogeny. The findings indicate that only the new bat coronavirus followed by pangolin coronaviruses have close evolutionary related with the novel SARS-CoV-2. The present study strongly supports that like the human host the coronavirus had undergone rapid evolution in bats and pangolin as an amplifying host (Figure 12).

Earlier research claimed that snakes or pangolins may be intermediate hosts for creating the coronavirus by recombination events [50]. Cross-species transmission of zoonotic coronaviruses (CoVs) can result in disease outbreaks [51]. Molecular analysis supported bats as natural hosts for SARS-CoV, but palm civets (*Paguma larvata*) had a critical role in the transmission to humans [52, 53]. Bats are implicated in SARS-CoV-2 origin. A very similar SARS-CoV-2 strain (RaTG13 CoV) was detected in *Rhinolophus affinis* bat with 96% genome similarity compared with SARS-CoV-2 genome sequence. Considering that bats were in hibernation when the outbreak occurred, the virus is more likely to have been transmitted via other species [54]. Both protein (9b and ORF14) genome phylogeny results of the present study are supported by the hypothesis for the zoonotic transmission route was constructed based on contact with Malayan pangolins (*Manis javanica*) by visitors of Huanan seafood market in Wuhan, China [55]. The close phylogenetic relationship to RaTG13 provides evidence that 2019-nCoV may have originated in bats [10]. Differently from bats, which are able to suppress viral replication, pangolin is an amplifying host which allows the increase of viral load and accelerated SARS-CoV-2 jump to human host and human-to-human transmission subsequently [56].

Another study, which supports the results of present finding, showed that the bat and pangolin coronaviruses were the most related to SARS-CoV-2 with 96% and 86% of identity all along the genome [57]. The comparison study from bat and pangolin by Li and his friends explains that BetaCoV/bat/Yunnan/RaTG13/2013 virus was more similar to the SARS-CoV-2 virus than the coronavirus obtained from the two pangolin samples (SRR10168377 and SRR10168378). The human SARS-CoV-2 virus, which is responsible for the recent outbreak of COVID-19, did not come directly from pangolins [13].

Tunnels are access paths connecting the interior of molecular systems with the surrounding environment. The presence of tunnels in proteins influences their reactivity, as they determine the nature and intensity of their interactions. Tunnel analysis of the newly predicted structures of the present study has estimated the presence of multiple tunnels in ORF14 protein. The *β* sheets of ORF9b form a tent-like structure which contains a 22 Å long central cavity, lined by hydrophobic side chains, which spans the molecule and is open at both ends [36]. The presence of multiple tunnels in this so far uncharacterized protein may take a key role in a large number of transport pathways for small ligands influencing their reactivity. It has been experimentally demonstrated that the tunnels and their properties can define many important protein characteristics like substrate specificity, enantioselectivity, stability, and activity [58]. The details of the structure verification report have been deposited to Modelarchive and will be available to download along with the structures (https://www.modelarchive.org/doi/10.xxxx/).

Several years before the outbreak of SARS, two other zoonotic viruses, Nipah virus and Hendra virus, emerged in Asia and Australia; they were both known to originate from bats [59, 60]. This led scientists to consider bats in the search for reservoirs of SARS-CoV. The present study on the evolution of 9b and ORF14 also highly indicates the bat origin for the newly emerged human SARS-CoV-2. Understanding the bat origin of human coronaviruses is helpful for the prediction and prevention of another pandemic emergence in the future [61].

In a recent correspondence published in Nature Medicine, Andersen et al. [16] clearly showed that SARS-CoV-2 is not a laboratory construct or a purposefully manipulated virus. The potential for close contact between bats, civets, and humans in the wildlife trade in southern China, coupled with a possible propensity of these bats to foster CoV host-shifts, could explain SARS-like CoVs as the source of SARS-CoV [8]. This potential supports molecular results on bat CoVs that suggest a recent host shift from bats to civets or other animals and humans [62]. A recent study also reported that the sequence of bat coronavirus RaTG13, sampled from a *Rhinolophus affinis* bat, is ~96% identical overall to SARS-CoV-2 [10]. With human activity increasingly overlapping the habitats of bats, disease outbreaks resulting from spillover of bat coronaviruses will continue to occur in the future, despite the fact that direct transmission of bat coronaviruses to humans appears to be rare [61].

It is reasonable to propose that ORF9b in SARS-CoV-2 may contribute to viral pathogenesis as I-protein in mouse hepatitis virus (MHV) does. The interactions between S, N, and ORF9b may help to localize ORF9b inside the particles but closer to the envelope. The structure of ORF9b, an intertwined dimer with an amphipathic outer surface and a long hydrophobic lipid binding tunnel, suggests how this protein may interact, via an unusual anchoring mechanism, with compartments of the ER-Golgi network to act as an accessory protein during the assembly of the SARS virion (Meier et al., 2006). However, further analyses of the properties and functions of ORF9b and ORF14 proteins are still necessary to understand its contribution to virus pathogenesis. All current studies on accessory proteins of coronaviruses, including SARS-CoV-2, suggest that they are not essential for virus replication [63] but do affect viral release, stability, and pathogenesis and finally contribute to virulence [64].

## 4. Conclusion

The RNA genome of SARS-CoV-2 has 29.9 kb nucleotides, encoding 14 open reading frames (ORFs) for 29 proteins, although one may not be expressed. Studying these different components of the virus as well as how they interact with human cells has already yielded some clues but much remains to be explored. The present study reported theoretical modeling, sequence-based, and structure-based functional characterization of two accessory protein-9b and ORF14 of SARS-CoV-2 p. Phylogenetic analysis of both proteins revealed a close evolutionary relationship between the newly emerged human SARS-CoV-2 and bat SARS-like corona virus. The whole-genome phylogeny indicates that 2019-nCoV may have originated in bat, undergone rapid evolution in bats, and pangolin may more likely to be an amplifying host. The presence of a large number of tunnels in the 9b protein indicates its high reactivity. The theoretical structures and statistical verification reports were successfully deposited in the Model Archive. The theoretical structures would perhaps be useful for advanced computational analysis of interactions of each protein for detailed functional analysis, understanding of viral pathogenesis and virulence for structure-based drug design, or to study potential vaccines, if at all, towards to prevent epidemics and pandemics in the absence of a complete experimental structure.

## Figures and Tables

**Table 1:** BLAST results against available PDB structures for selection of the modeling method, template selection for the structures of 9b and ORF14 proteins.

Sl no.	Protein name and UniProtKB accession number	Length (aa residue)	PDB Template (S)	Identity with template (%)	E-value	Query coverage	The final structure/modeling method selected
1	ORF9b protein (P0DTD2)	97	2CME_B (79 aa)	70.93%	2*e*-34	89%	Comparative modelling
2	ORF14 protein (P0DTD3)	73	3A32_A	39.13%	9.0	31%	*Ab initio* modelling

**Table 2:** Physicochemical parameters of SARS-CoV-2 9b and ORF14 proteins and comparison with other coronaviruses.

Protein name	UniProt KB accession no. organism	Length	MW (Da)	pI	Chemical formula	Instability index	Aliphatic index	Gravy^∗^
ORF9b protein	P0DTD2|human SARS-CoV-2	97	10796.66	6.56	C_478_H_796_N_130_O_142_S_5_	33.11	105.46	-0.085
	Q3LZX3|bat CoV	97	10722.54	6.05	C_475_H_786_N_128_O_142_S_5_	41.80	104.43	-0.012
	Q6RD12|human SARS-CoV	98	10802.45	4.90	C_472_H_778_N_130_O_148_S_5_	38.95	98.47	-0.122
	A0A023PUR2|*R. affinis* CoV	98	10781.50	5.69	C_475_H_787_N_131_O_145_S_4_	39.97	105.41	-0.050
	Tr|Q3ZTD0|SARS-CoV civet010	98	10790.40	4.90	C_470_H_774_N_130_O_149_S_5_	38.95	94.49	-0.176
ORF14 protein	P0DTD3|human SARS-CoV-2	73	8049.65	5.79	C_359_H_588_N_92_O_100_S_8_	32.56	125.62	0.603
	AVP78040|bat CoV	70	7690.13	6.38	C_341_H_555_N_91_O_96_S_7_	25.59	117.14	0.466
	ARO76392|human SARS-CoV	70	7842.29	6.25	C_354_H_571_N_93_O_97_S_5_	32.40	119.86	0.321
	AAU04674|SARS-CoV civet	70	7868.37	6.25	C_357_H_577_N_93_O_96_S_5_	23.92	125.43	0.387
	AHX37568|*R. affinis* CoV	70	7810.21	6.39	C_352_H_563_N_93_O_97_S_5_	32.81	111.57	0.196

^∗^GRAVY: Grand average of hydropathicity.

**Table 3:** Predicted functions of SARS-CoV-2 9b and ORF14 proteins with respective ProFunc score (shown within parenthesis).

Protein name	Summary of predicted function			
	Protein name terms	Gene ontology (GO) terms		
		Cellular component	Biological process	Biochemical function
9b protein	SARS coronavirus ORF9b (0.90) aquifex aeolicus trbp111 structure-specific (0.50) aeolicus trbp111 structure-specific trna (0.50) trbp111 structure-specific trna binding (0.50) ustilago maydis lipase um03410 (0.50) maydis lipase um03410 short (0.50) lipase um03410 short form (0.50) um03410 short form without (0.50)	Extracellular region (1.33) cytoplasm (0.85)	Cellular process (1.66) cellular metabolic process (1.66)	tRNA binding (0.50) RNA binding (0.50) aminoacyl\-tRNA ligase activity (0.50) binding (0.50)
ORF14 protein	Human (1.72) domain (1.56) atcc (1.00) nmr (1.00) aminoimidazole riboside kinase (0.70) phycocyanin (0.57) ccm3 (0.50) c-terminal regulatory domain stk25 (0.50)	Cytoplasm (1.86) cell (1.86) cell part (1.86) intracellular (1.86)	Metabolic process (3.06) cellular process (2.27) cellular metabolic process (2.27) primary metabolic process (1.53)	Catalytic activity (2.91) binding (2.48) metal ion binding (1.61) ion binding (1.61)

## Data Availability

(1) The resultant protein structures are deposited in ModelArchive (https://www.modelarchive.org/). The same data has been provided in a supplementary file (folder name: Data availability). (2) The supplementary file for the data generated in the project has been deposited to ChemRxiv. Preprint. doi:10.26434/chemrxiv.12424958.v1 (supplementary file). (3) All the above data are also included along with this manuscript.

## Additional Points

*Commercial Declarations*. It is made available under a CC-BY-NC-ND 4.0 International license.

## Supplementary Materials

Supplementary Materials Table S1: computed cavities in the 3D structure of ORF9b protein for active sites. Table S2: computed cavities in the 3D structure of ORF14 protein for active sites. Figure S1: secondary structure profile of 9b protein. Figure S2: secondary structure profile of ORF14 protein. Figure S3: QMEANDisCo local quality estimate for 9b protein. Figure S4: QMEANDisCo local quality estimate for ORF14 protein. Figure S5: protein 9b structure verification in ERRAT. Figure S6: protein ORF14 structure verification in ERRAT. Figure S7: profile of tunnel 1 in 9b protein. Figure S8: profile of tunnel 2 in 9b protein. Figure S9: tunnel-profile of ORF14 protein. Figure S10: hydropathicity plot for 9b protein. Figure S11: hydrophobicity plot for ORF14 protein. Annexure 1: protein 9b structure verification. Annexure 2: ORF14 protein structure verification.
