# Supplementary material for: Sequence Analysis and Structure Prediction of SARS-CoV-2 Accessory Proteins 9b and ORF14: Evolutionary Analysis Indicates Close Relatedness to Bat Coronavirus
Source: Biomed Res Int. 2020 Oct 20;2020:7234961. doi: 10.1155/2020/7234961 (PMC7576348; doi:10.1155/2020/7234961)
Supplement: Supplementary Materials — Table S1: computed cavities in the 3D structure of ORF9b protein for active sites. Table S2: computed cavities in the 3D structure of ORF14 protein for active sites. Figure S1: secondary structure profile of 9b protein. Figure S2: secondary structure profile of ORF14 protein. Figure S3: QMEANDisCo local quality estimate for 9b protein. Figure S4: QMEANDisCo local quality estimate for ORF14 protein. Figure S5: protein 9b structure verification in ERRAT. Figure S6: protein ORF14 structure verification in ERRAT. Figure S7: profile of tunnel 1 in 9b protein. Figure S8: profile of tunnel 2 in 9b protein. Figure S9: tunnel-profile of ORF14 protein. Figure S10: hydropathicity plot for 9b protein. Figure S11: hydrophobicity plot for ORF14 protein. Annexure 1: protein 9b structure verification. Annexure 2: ORF14 protein structure verification. [file 7234961.f1.zip › Annexure 1_Protein 9b structure verification.rtf]

Protein 9b structure verification report
Verification was carried out in QMEANDisCo 4.0.0  
QMEANDisCo 4.0.0  Global Score: 0.67 ± 0.09
 "scores": {
        "global_scores": {
          "acc_agreement_norm_score": 0.6494845361,
          "acc_agreement_z_score": 0.1617901027,
          "avg_local_score": 0.6680036794,
          "avg_local_score_error": 0.088,
          "cbeta_norm_score": -0.0056014962,
          "cbeta_z_score": -1.3301821032,
          "interaction_norm_score": -0.0027754459,
          "interaction_z_score": -2.6474670371,
          "packing_norm_score": -0.182711631,
          "packing_z_score": -2.0596710557,
          "qmean4_norm_score": 0.7025304037,
          "qmean4_z_score": -1.6461424389000001,
          "qmean6_norm_score": 0.6732314904000001,
          "qmean6_z_score": -1.9616206465000001,
          "ss_agreement_norm_score": 0.2885876045,
          "ss_agreement_z_score": -2.0492572678,
          "torsion_norm_score": -0.3015871221,
          "torsion_z_score": -0.37360978770000003
        },
        "local_scores": {
          "A": [
            0.6575159699,
            0.6437379421,
            0.7581244033000001,
            0.7563726296000001,
            0.7781746339000001,
            0.7294501104000001,
            0.7118361253000001,
            0.6398691825,
            0.6776970473,
            0.7594022785000001,
            0.6648354703,
            0.7218106212000001,
            0.7513195075,
            0.8472636965,
            0.8731248108,
            0.7932749633,
            0.817797697,
            0.7617110475000001,
            0.7965483817,
            0.7761359229,
            0.8044700528000001,
            0.7751325541,
            0.7390442977,
            0.650231799,
            0.4798302715,
            0.24619251320000002,
            0.2580592858,
            0.2683330299,
            0.2836639436,
            0.2718224063,
            0.26973416510000003,
            0.2621861103,
            0.3112198426,
            0.28410784,
            0.2811467119,
            0.2946913053,
            0.4212517745,
            0.4981148714,
            0.7166620250000001,
            0.7223573125,
            0.7937607506000001,
            0.8075945005,
            0.8645410565,
            0.8583513896,
            0.8565241673,
            0.8268882525,
            0.7521975118,
            0.7433691567,
            0.8092398256000001,
            0.7823987728,
            0.7874509898000001,
            0.7623037758000001,
            0.6945721494,
            0.6618322317,
            0.6270534319000001,
            0.6662468296,
            0.7168878217,
            0.5776176728,
            0.6437488499,
            0.577638131,
            0.5168949056000001,
            0.5320536157,
            0.6264544902,
            0.4415527349,
            0.4706515658,
            0.5431250148,
            0.44102225640000003,
            0.6807854749000001,
            0.5686984964,
            0.6239387248,
            0.7384337507000001,
            0.7693102692,
            0.7628330106000001,
            0.7103507999,
            0.6443991038,
            0.7740300849,
            0.7808373458000001,
            0.7759225128,
            0.7935441509000001,
            0.7013850661000001,
            0.7232947017,
            0.7222478288,
            0.7621994024000001,
            0.7469372411,
            0.7512667646000001,
            0.7506342049,
            0.8054417469,
            0.7831990790000001,
            0.8018792426,
            0.7805988999,
            0.8448283644,
            0.8862346857000001,
            0.8661018853,
            0.8477978304,
            0.8205196637000001,
            0.7947974233,
            0.6476827389
          ]
