# Supplementary material for: Sequence Analysis and Structure Prediction of SARS-CoV-2 Accessory Proteins 9b and ORF14: Evolutionary Analysis Indicates Close Relatedness to Bat Coronavirus
Source: Biomed Res Int. 2020 Oct 20;2020:7234961. doi: 10.1155/2020/7234961 (PMC7576348; doi:10.1155/2020/7234961)
Supplement: Supplementary Materials — Table S1: computed cavities in the 3D structure of ORF9b protein for active sites. Table S2: computed cavities in the 3D structure of ORF14 protein for active sites. Figure S1: secondary structure profile of 9b protein. Figure S2: secondary structure profile of ORF14 protein. Figure S3: QMEANDisCo local quality estimate for 9b protein. Figure S4: QMEANDisCo local quality estimate for ORF14 protein. Figure S5: protein 9b structure verification in ERRAT. Figure S6: protein ORF14 structure verification in ERRAT. Figure S7: profile of tunnel 1 in 9b protein. Figure S8: profile of tunnel 2 in 9b protein. Figure S9: tunnel-profile of ORF14 protein. Figure S10: hydropathicity plot for 9b protein. Figure S11: hydrophobicity plot for ORF14 protein. Annexure 1: protein 9b structure verification. Annexure 2: ORF14 protein structure verification. [file 7234961.f1.zip › Annexure 2_ORF 14 protein_structure verification.rtf]

Structure verification report of ORF 14 protein 
Verification was carried out in QMEANDisCo 4.0.0  

QMEANDisCo 4.0.0 Global Score: 0.52 ± 0.11

 "scores": {
        "global_scores": {
          "acc_agreement_norm_score": 0.6301369863,
          "acc_agreement_z_score": -0.4546248242,
          "avg_local_score": 0.5161492531,
          "avg_local_score_error": 0.105,
          "cbeta_norm_score": -0.0043738712,
          "cbeta_z_score": -1.5119062247000001,
          "interaction_norm_score": -0.0218334172,
          "interaction_z_score": -0.7923254693,
          "packing_norm_score": -0.40294158150000003,
          "packing_z_score": 0.007814732000000001,
          "qmean4_norm_score": 0.7200594997,
          "qmean4_z_score": -1.1889773371,
          "qmean6_norm_score": 0.7036521102000001,
          "qmean6_z_score": -1.2472493282000001,
          "ss_agreement_norm_score": 0.562695838,
          "ss_agreement_z_score": -0.44020180600000003,
          "torsion_norm_score": -0.1916014419,
          "torsion_z_score": -0.7691830711000001
        },
