# Supplementary material for: Sequence Analysis and Structure Prediction of SARS-CoV-2 Accessory Proteins 9b and ORF14: Evolutionary Analysis Indicates Close Relatedness to Bat Coronavirus
Source: Biomed Res Int. 2020 Oct 20;2020:7234961. doi: 10.1155/2020/7234961 (PMC7576348; doi:10.1155/2020/7234961)
Supplement: Supplementary Materials — Table S1: computed cavities in the 3D structure of ORF9b protein for active sites. Table S2: computed cavities in the 3D structure of ORF14 protein for active sites. Figure S1: secondary structure profile of 9b protein. Figure S2: secondary structure profile of ORF14 protein. Figure S3: QMEANDisCo local quality estimate for 9b protein. Figure S4: QMEANDisCo local quality estimate for ORF14 protein. Figure S5: protein 9b structure verification in ERRAT. Figure S6: protein ORF14 structure verification in ERRAT. Figure S7: profile of tunnel 1 in 9b protein. Figure S8: profile of tunnel 2 in 9b protein. Figure S9: tunnel-profile of ORF14 protein. Figure S10: hydropathicity plot for 9b protein. Figure S11: hydrophobicity plot for ORF14 protein. Annexure 1: protein 9b structure verification. Annexure 2: ORF14 protein structure verification. [file 7234961.f1.zip › Table S1_computed cavities for active sites_9b.docx]

**Table S1.** Computed cavities in the 3D structure of Orf9b protein for active sites

| **cavity_1_NPQVDKGEYTAMIFRLS**  Cavity point 10.606, -3.416, -5.832  Volume of the Cavity = 1261 | **cavity_2_DKQPRVELNTFYIAM**  Cavity point 10.014, -0.066, 5.372  Volume of the Cavity = 971 |
| --- | --- |
| **cavity_3_QDENPKRVLTFYMI**  Cavity point -1.021 2.371 0.030  Volume of the Cavity = 734 | **cavity_4_QNPGVDKTEAIMLS**  Cavity point 3.847 -2.386 -7.661  Volume of the Cavity = 627 |
| **cavity_5_QPDTLKMAIVRSG**  Cavity point 8.366 9.481 -8.844  Volume of the Cavity = 586 | **cavity_6_ETLDPAFIKVRMH**  Cavity point 8.530 10.403 8.854  Volume of the Cavity = 561 |
| **cavity_7_TDLQKAMISVREPHG**  Cavity point 11.107 15.632 -3.355  Volume of the Cavity = 545 | **cavity_8_DPLEFTYIVKMARHG**  Cavity point 12.674 6.217 6.615  Volume of the Cavity = 521 |
| **cavity_9_ETPADLQKMISR**  Cavity point 4.850 14.991 -1.592  Volume of the Cavity = 276 |  |
