# Supplementary material for: Sequence Analysis and Structure Prediction of SARS-CoV-2 Accessory Proteins 9b and ORF14: Evolutionary Analysis Indicates Close Relatedness to Bat Coronavirus
Source: Biomed Res Int. 2020 Oct 20;2020:7234961. doi: 10.1155/2020/7234961 (PMC7576348; doi:10.1155/2020/7234961)
Supplement: Supplementary Materials — Table S1: computed cavities in the 3D structure of ORF9b protein for active sites. Table S2: computed cavities in the 3D structure of ORF14 protein for active sites. Figure S1: secondary structure profile of 9b protein. Figure S2: secondary structure profile of ORF14 protein. Figure S3: QMEANDisCo local quality estimate for 9b protein. Figure S4: QMEANDisCo local quality estimate for ORF14 protein. Figure S5: protein 9b structure verification in ERRAT. Figure S6: protein ORF14 structure verification in ERRAT. Figure S7: profile of tunnel 1 in 9b protein. Figure S8: profile of tunnel 2 in 9b protein. Figure S9: tunnel-profile of ORF14 protein. Figure S10: hydropathicity plot for 9b protein. Figure S11: hydrophobicity plot for ORF14 protein. Annexure 1: protein 9b structure verification. Annexure 2: ORF14 protein structure verification. [file 7234961.f1.zip › Table S2_computed cavities for active sites_ORF14.docx]

**Table S2.** Computed cavities in the 3D structure of Orf14 protein for active sites

| [**cavity_1_HEPIATVLKWCDMY**](http://www.scfbio-iitd.res.in/dock/48091783ACTIVE/cavity_1_HEPIATVLKWCDMY)  Cavity point -7.341 17.615 -5.427  Volume of the Cavity = 662 | [**cavity_2_PATIHQVLWKYENMCSF**](http://www.scfbio-iitd.res.in/dock/48091783ACTIVE/cavity_2_PATIHQVLWKYENMCSF)  Cavity point -1.634 12.400 3.280  Volume of the Cavity = 494 |
| --- | --- |
| [**cavity_3_EHPIATQVLWKYMNCS**](http://www.scfbio-iitd.res.in/dock/48091783ACTIVE/cavity_3_EHPIATQVLWKYMNCS)  Cavity point -5.398 8.710 3.211  Volume of the Cavity = 405 | [**cavity_4_PTAIHQVLWKCMYNE**](http://www.scfbio-iitd.res.in/dock/48091783ACTIVE/cavity_4_PTAIHQVLWKCMYNE)  Cavity point -1.878 15.931 -1.260  Volume of the Cavity = 393 |
| **cavity_5_WQLVAYMEKCSF**  Cavity point 4.898 10.429 -5.332  Volume of the Cavity = 391 | **cavity_6_EITAVPLWM**  Cavity point -7.174 12.490 -9.100  Volume of the Cavity = 342 |
| **cavity_7_HPTAVLKDCEYG**  Cavity point -6.500 21.177 2.500  Volume of the Cavity = 311 | **cavity_8_EAQIVYLMNCS**  Cavity point -3.291 2.648 0.586  Volume of the Cavity = 280 |
| **cavity_9_VLKPDAYENHQCF**  Cavity point 1.319 19.261 7.740  Volume of the Cavity = 260 | [**cavity_10_LCAMGVHQSK**](http://www.scfbio-iitd.res.in/dock/48091783ACTIVE/cavity_10_LCAMGVHQSK)  Cavity point 2.905 22.194 -4.904  Volume of the Cavity = 97 |
