# Supplementary figures and images for: Sequence Analysis and Structure Prediction of SARS-CoV-2 Accessory Proteins 9b and ORF14: Evolutionary Analysis Indicates Close Relatedness to Bat Coronavirus
Source: Biomed Res Int. 2020 Oct 20;2020:7234961. doi: 10.1155/2020/7234961 (PMC7576348; doi:10.1155/2020/7234961)

## Slide 1
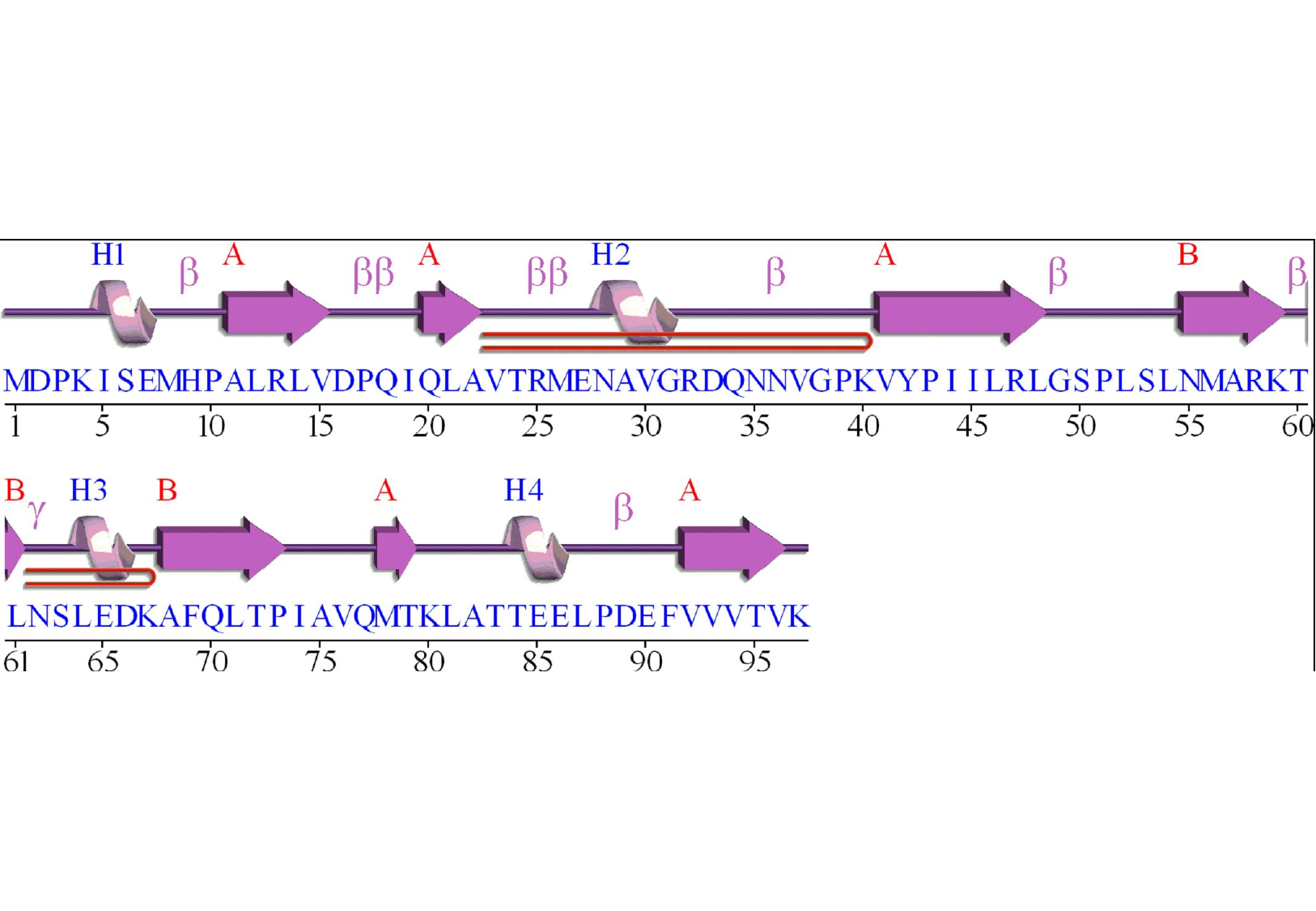

Supplement: Supplementary Materials — Table S1: computed cavities in the 3D structure of ORF9b protein for active sites. Table S2: computed cavities in the 3D structure of ORF14 protein for active sites. Figure S1: secondary structure profile of 9b protein. Figure S2: secondary structure profile of ORF14 protein. Figure S3: QMEANDisCo local quality estimate for 9b protein. Figure S4: QMEANDisCo local quality estimate for ORF14 protein. Figure S5: protein 9b structure verification in ERRAT. Figure S6: protein ORF14 structure verification in ERRAT. Figure S7: profile of tunnel 1 in 9b protein. Figure S8: profile of tunnel 2 in 9b protein. Figure S9: tunnel-profile of ORF14 protein. Figure S10: hydropathicity plot for 9b protein. Figure S11: hydrophobicity plot for ORF14 protein. Annexure 1: protein 9b structure verification. Annexure 2: ORF14 protein structure verification. [file 7234961.f1.zip › Figure S1_orf 9b_secondary structure.pptx]

## Slide 1
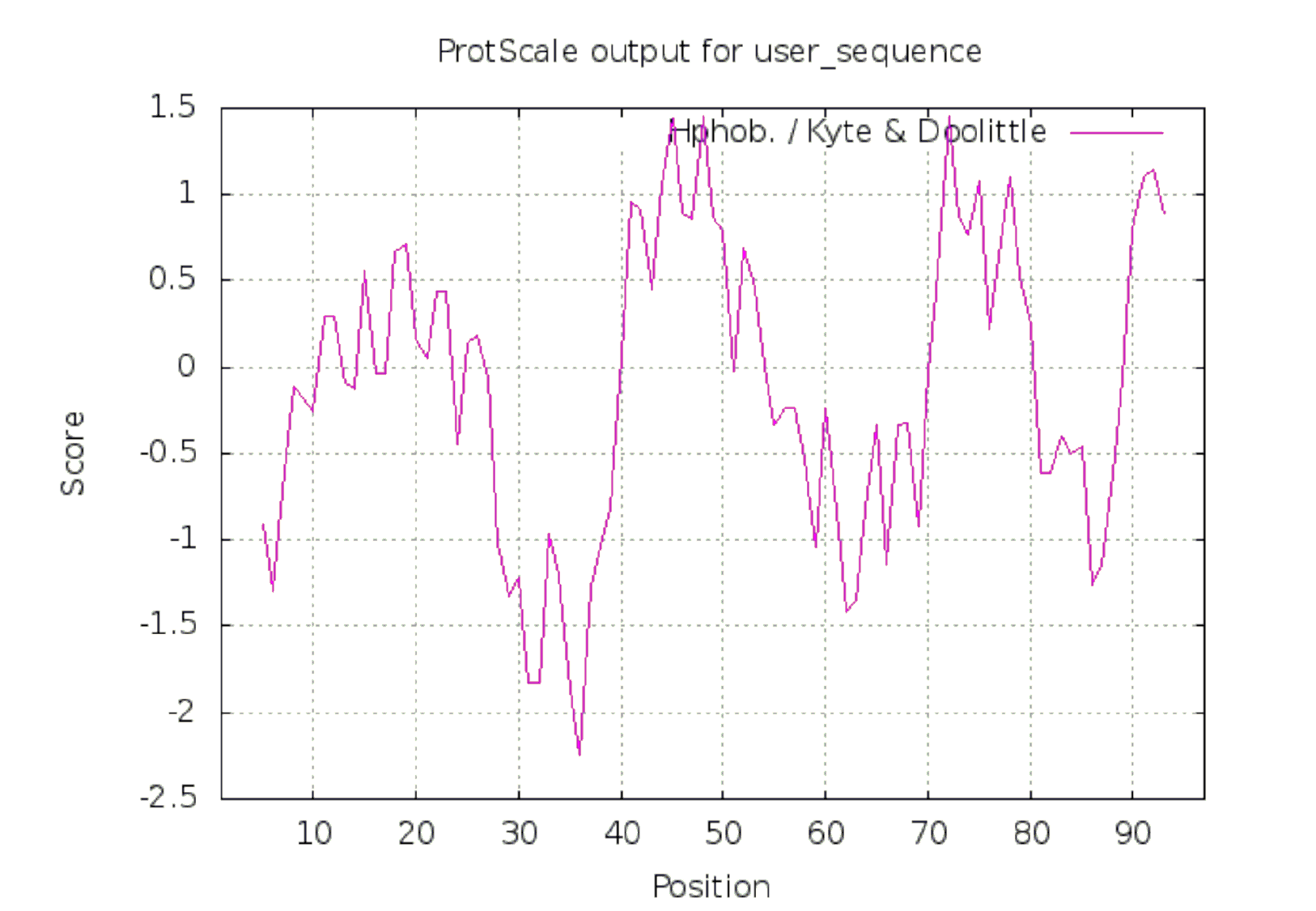

Supplement: Supplementary Materials — Table S1: computed cavities in the 3D structure of ORF9b protein for active sites. Table S2: computed cavities in the 3D structure of ORF14 protein for active sites. Figure S1: secondary structure profile of 9b protein. Figure S2: secondary structure profile of ORF14 protein. Figure S3: QMEANDisCo local quality estimate for 9b protein. Figure S4: QMEANDisCo local quality estimate for ORF14 protein. Figure S5: protein 9b structure verification in ERRAT. Figure S6: protein ORF14 structure verification in ERRAT. Figure S7: profile of tunnel 1 in 9b protein. Figure S8: profile of tunnel 2 in 9b protein. Figure S9: tunnel-profile of ORF14 protein. Figure S10: hydropathicity plot for 9b protein. Figure S11: hydrophobicity plot for ORF14 protein. Annexure 1: protein 9b structure verification. Annexure 2: ORF14 protein structure verification. [file 7234961.f1.zip › Figure S10_orf9b Hydrophobicity plot.pptx]

## Slide 1
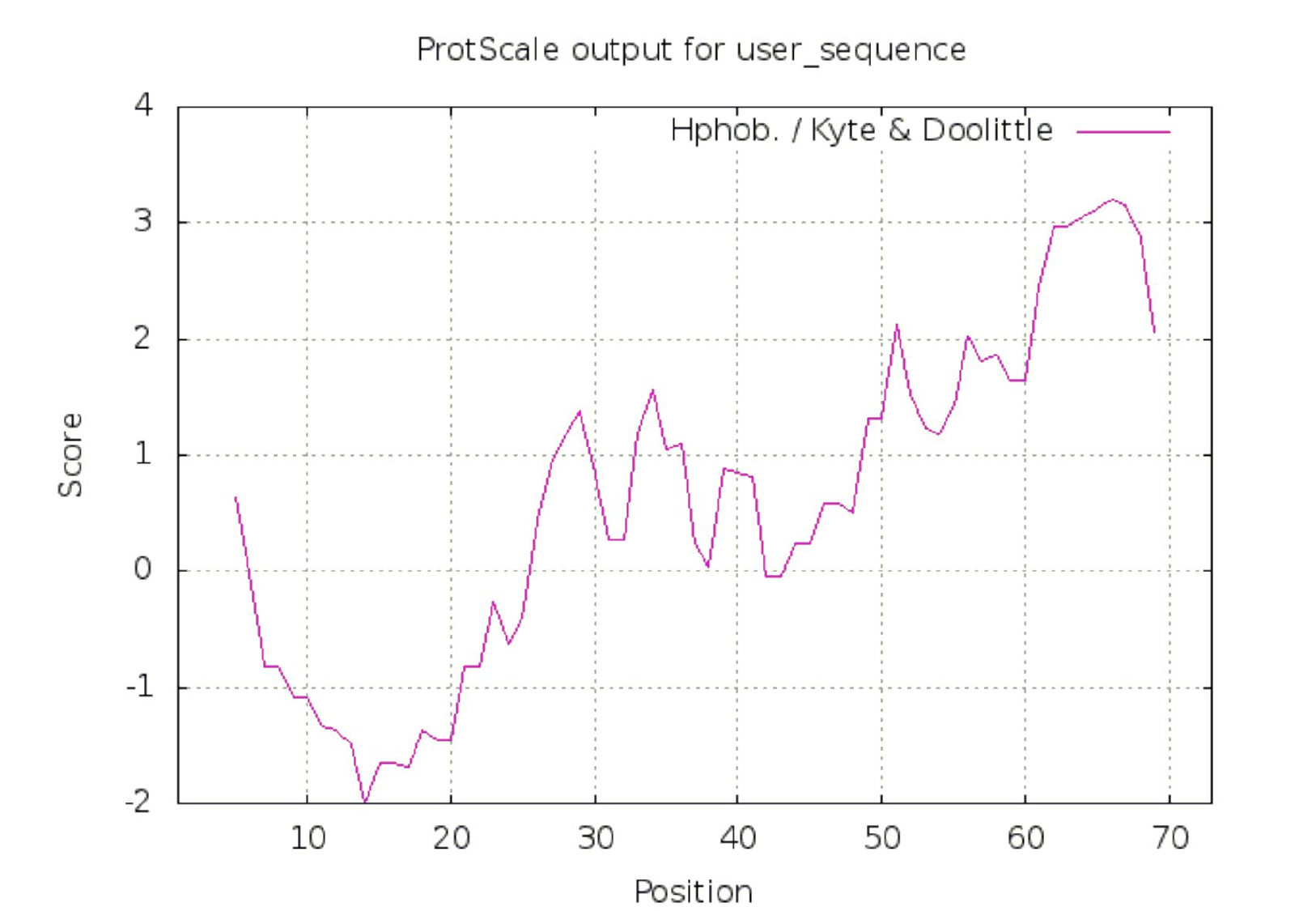

Supplement: Supplementary Materials — Table S1: computed cavities in the 3D structure of ORF9b protein for active sites. Table S2: computed cavities in the 3D structure of ORF14 protein for active sites. Figure S1: secondary structure profile of 9b protein. Figure S2: secondary structure profile of ORF14 protein. Figure S3: QMEANDisCo local quality estimate for 9b protein. Figure S4: QMEANDisCo local quality estimate for ORF14 protein. Figure S5: protein 9b structure verification in ERRAT. Figure S6: protein ORF14 structure verification in ERRAT. Figure S7: profile of tunnel 1 in 9b protein. Figure S8: profile of tunnel 2 in 9b protein. Figure S9: tunnel-profile of ORF14 protein. Figure S10: hydropathicity plot for 9b protein. Figure S11: hydrophobicity plot for ORF14 protein. Annexure 1: protein 9b structure verification. Annexure 2: ORF14 protein structure verification. [file 7234961.f1.zip › Figure S11_orf14_Hydrophobicity plot.pptx]

## Slide 1
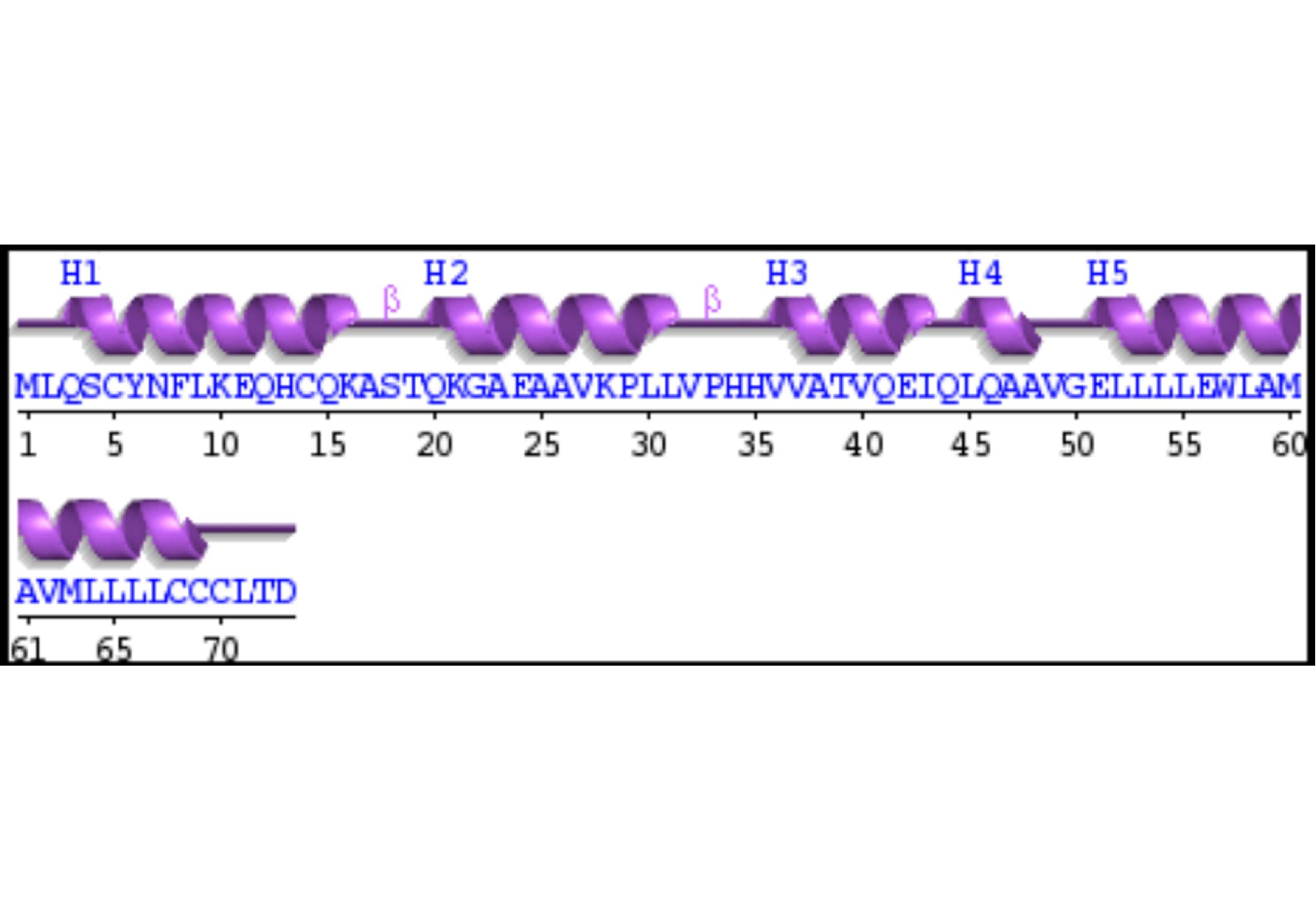

Supplement: Supplementary Materials — Table S1: computed cavities in the 3D structure of ORF9b protein for active sites. Table S2: computed cavities in the 3D structure of ORF14 protein for active sites. Figure S1: secondary structure profile of 9b protein. Figure S2: secondary structure profile of ORF14 protein. Figure S3: QMEANDisCo local quality estimate for 9b protein. Figure S4: QMEANDisCo local quality estimate for ORF14 protein. Figure S5: protein 9b structure verification in ERRAT. Figure S6: protein ORF14 structure verification in ERRAT. Figure S7: profile of tunnel 1 in 9b protein. Figure S8: profile of tunnel 2 in 9b protein. Figure S9: tunnel-profile of ORF14 protein. Figure S10: hydropathicity plot for 9b protein. Figure S11: hydrophobicity plot for ORF14 protein. Annexure 1: protein 9b structure verification. Annexure 2: ORF14 protein structure verification. [file 7234961.f1.zip › Figure S2_ORF 14 secondary structure.pptx]

## Slide 1
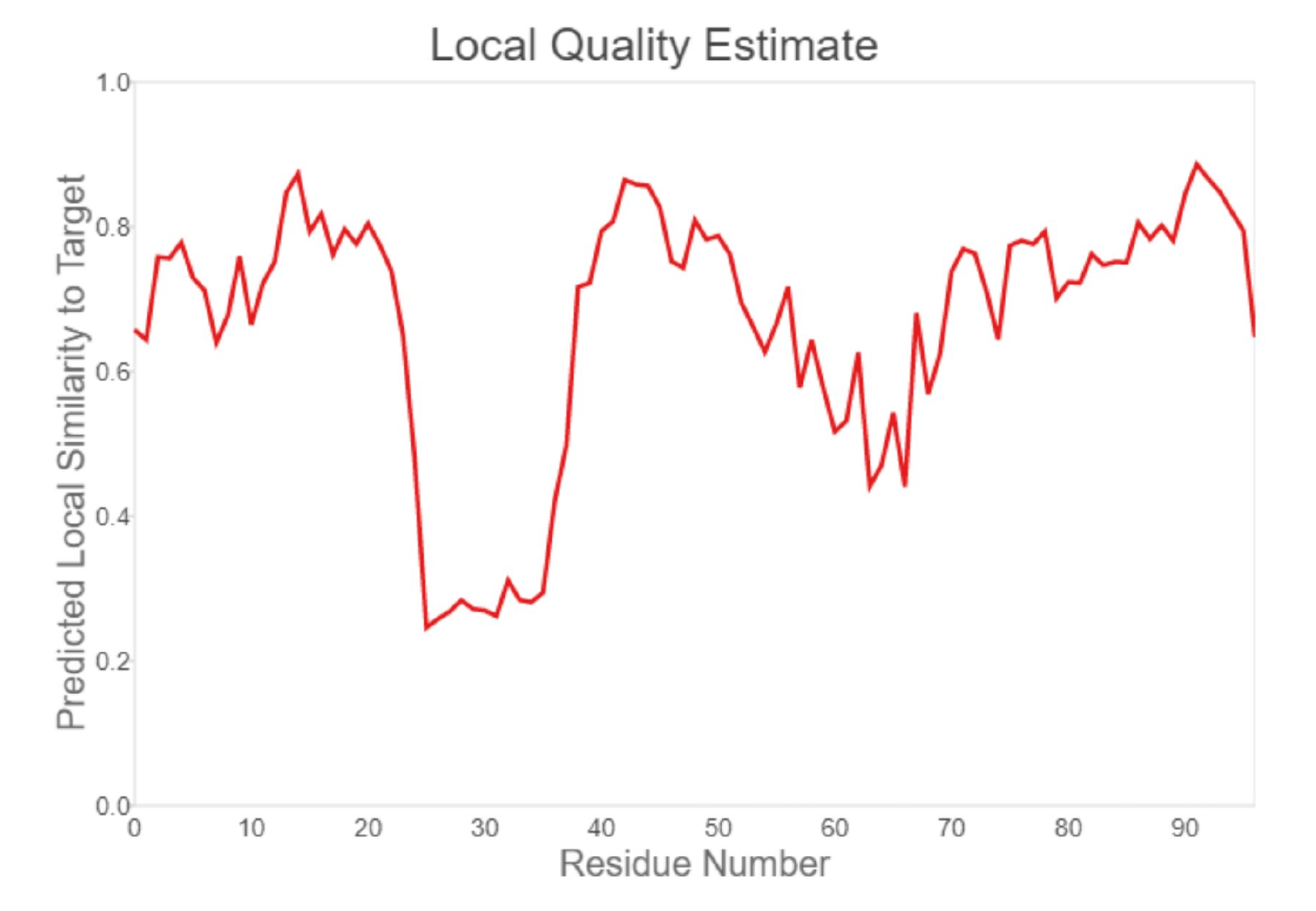

Supplement: Supplementary Materials — Table S1: computed cavities in the 3D structure of ORF9b protein for active sites. Table S2: computed cavities in the 3D structure of ORF14 protein for active sites. Figure S1: secondary structure profile of 9b protein. Figure S2: secondary structure profile of ORF14 protein. Figure S3: QMEANDisCo local quality estimate for 9b protein. Figure S4: QMEANDisCo local quality estimate for ORF14 protein. Figure S5: protein 9b structure verification in ERRAT. Figure S6: protein ORF14 structure verification in ERRAT. Figure S7: profile of tunnel 1 in 9b protein. Figure S8: profile of tunnel 2 in 9b protein. Figure S9: tunnel-profile of ORF14 protein. Figure S10: hydropathicity plot for 9b protein. Figure S11: hydrophobicity plot for ORF14 protein. Annexure 1: protein 9b structure verification. Annexure 2: ORF14 protein structure verification. [file 7234961.f1.zip › Figure S3_9b_Local_quality_estimate.pptx]

## Slide 1
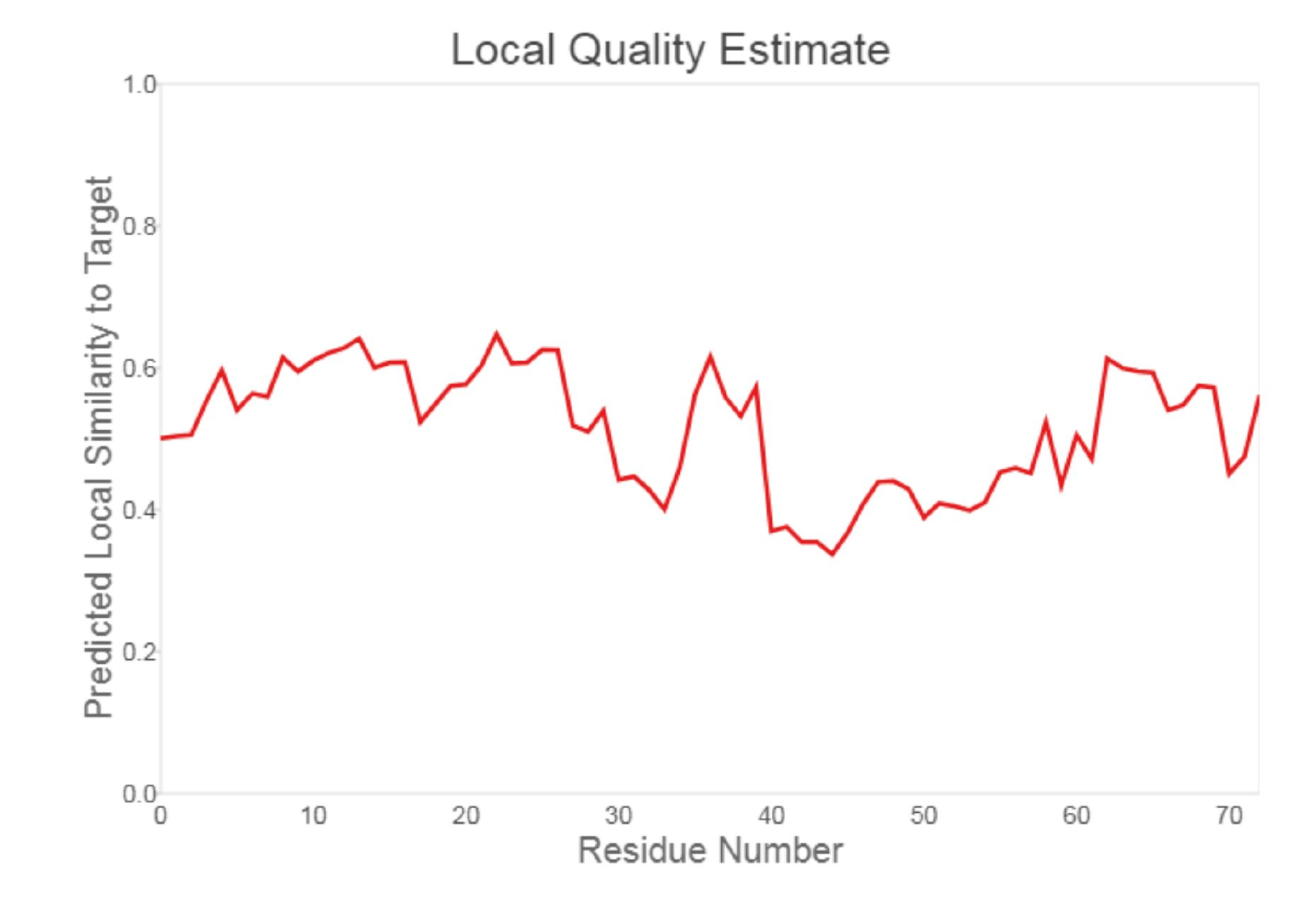

Supplement: Supplementary Materials — Table S1: computed cavities in the 3D structure of ORF9b protein for active sites. Table S2: computed cavities in the 3D structure of ORF14 protein for active sites. Figure S1: secondary structure profile of 9b protein. Figure S2: secondary structure profile of ORF14 protein. Figure S3: QMEANDisCo local quality estimate for 9b protein. Figure S4: QMEANDisCo local quality estimate for ORF14 protein. Figure S5: protein 9b structure verification in ERRAT. Figure S6: protein ORF14 structure verification in ERRAT. Figure S7: profile of tunnel 1 in 9b protein. Figure S8: profile of tunnel 2 in 9b protein. Figure S9: tunnel-profile of ORF14 protein. Figure S10: hydropathicity plot for 9b protein. Figure S11: hydrophobicity plot for ORF14 protein. Annexure 1: protein 9b structure verification. Annexure 2: ORF14 protein structure verification. [file 7234961.f1.zip › Figure S4_ORF14_Local_quality_estimate.pptx]

## Slide 1
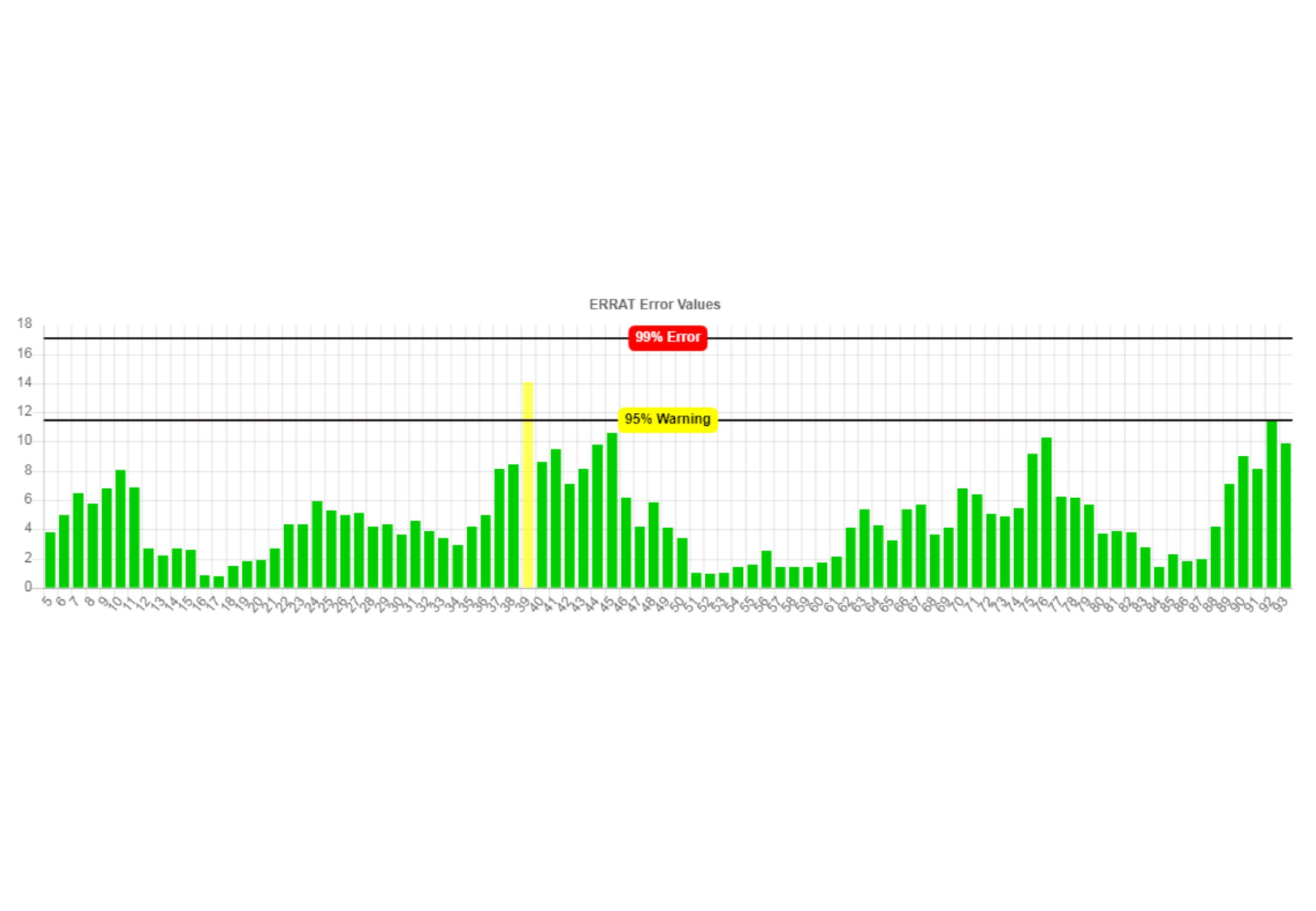

Supplement: Supplementary Materials — Table S1: computed cavities in the 3D structure of ORF9b protein for active sites. Table S2: computed cavities in the 3D structure of ORF14 protein for active sites. Figure S1: secondary structure profile of 9b protein. Figure S2: secondary structure profile of ORF14 protein. Figure S3: QMEANDisCo local quality estimate for 9b protein. Figure S4: QMEANDisCo local quality estimate for ORF14 protein. Figure S5: protein 9b structure verification in ERRAT. Figure S6: protein ORF14 structure verification in ERRAT. Figure S7: profile of tunnel 1 in 9b protein. Figure S8: profile of tunnel 2 in 9b protein. Figure S9: tunnel-profile of ORF14 protein. Figure S10: hydropathicity plot for 9b protein. Figure S11: hydrophobicity plot for ORF14 protein. Annexure 1: protein 9b structure verification. Annexure 2: ORF14 protein structure verification. [file 7234961.f1.zip › Figure S5_Structure of 9b_ERRAT verification.pptx]

## Slide 1
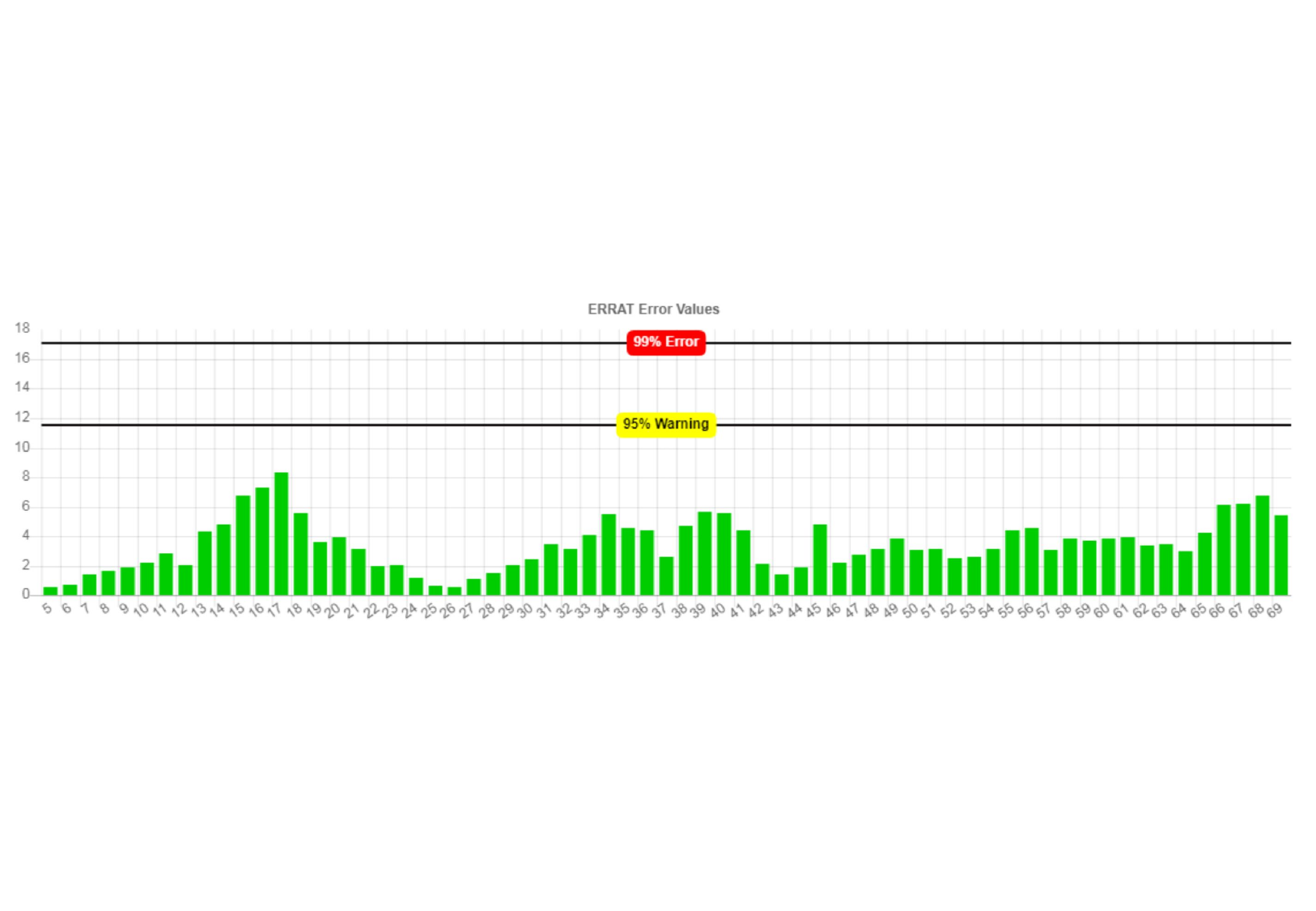

Supplement: Supplementary Materials — Table S1: computed cavities in the 3D structure of ORF9b protein for active sites. Table S2: computed cavities in the 3D structure of ORF14 protein for active sites. Figure S1: secondary structure profile of 9b protein. Figure S2: secondary structure profile of ORF14 protein. Figure S3: QMEANDisCo local quality estimate for 9b protein. Figure S4: QMEANDisCo local quality estimate for ORF14 protein. Figure S5: protein 9b structure verification in ERRAT. Figure S6: protein ORF14 structure verification in ERRAT. Figure S7: profile of tunnel 1 in 9b protein. Figure S8: profile of tunnel 2 in 9b protein. Figure S9: tunnel-profile of ORF14 protein. Figure S10: hydropathicity plot for 9b protein. Figure S11: hydrophobicity plot for ORF14 protein. Annexure 1: protein 9b structure verification. Annexure 2: ORF14 protein structure verification. [file 7234961.f1.zip › Figure S6_Structure of ORF14_ERRAT verification.pptx]

## Slide 1
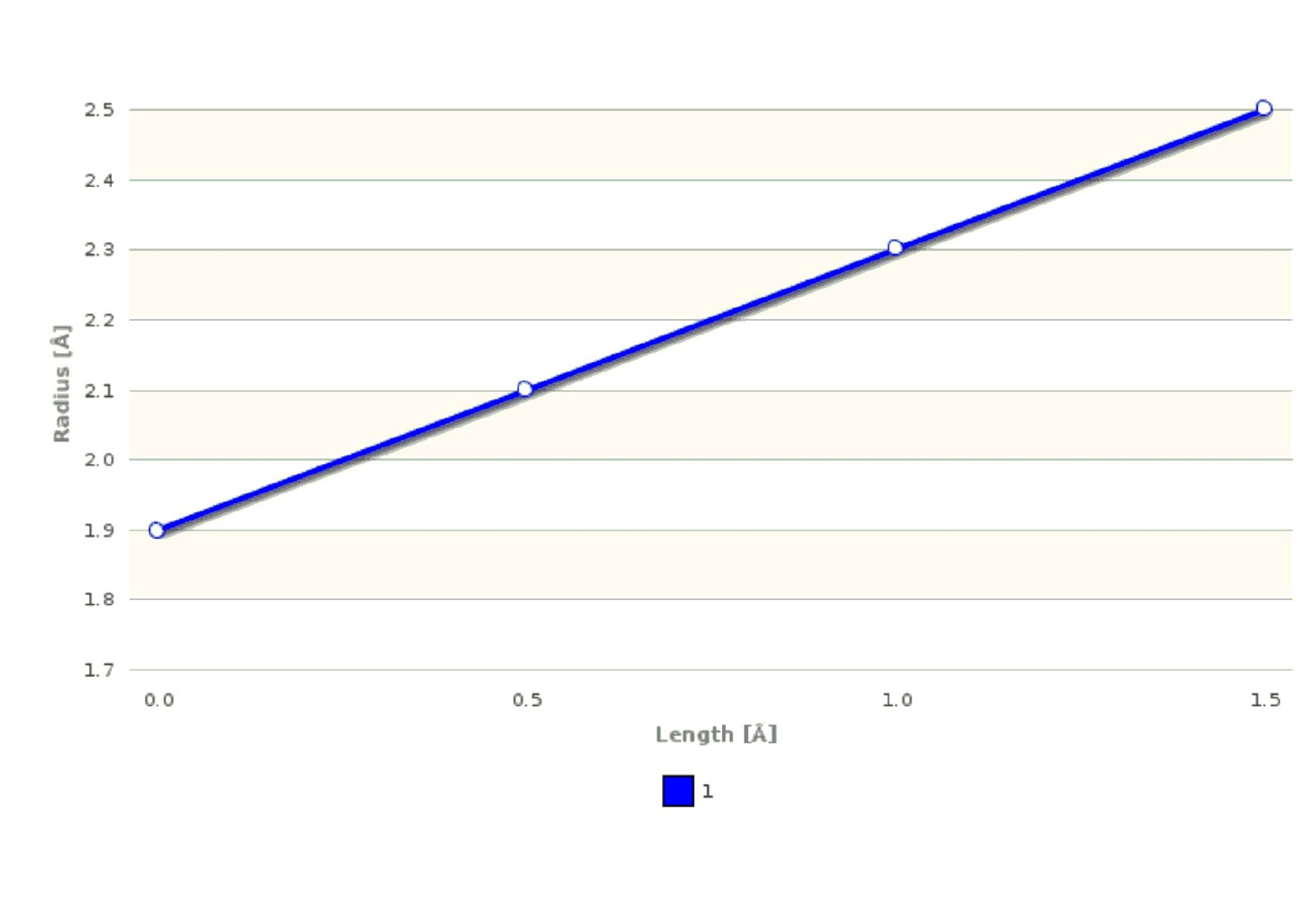

Supplement: Supplementary Materials — Table S1: computed cavities in the 3D structure of ORF9b protein for active sites. Table S2: computed cavities in the 3D structure of ORF14 protein for active sites. Figure S1: secondary structure profile of 9b protein. Figure S2: secondary structure profile of ORF14 protein. Figure S3: QMEANDisCo local quality estimate for 9b protein. Figure S4: QMEANDisCo local quality estimate for ORF14 protein. Figure S5: protein 9b structure verification in ERRAT. Figure S6: protein ORF14 structure verification in ERRAT. Figure S7: profile of tunnel 1 in 9b protein. Figure S8: profile of tunnel 2 in 9b protein. Figure S9: tunnel-profile of ORF14 protein. Figure S10: hydropathicity plot for 9b protein. Figure S11: hydrophobicity plot for ORF14 protein. Annexure 1: protein 9b structure verification. Annexure 2: ORF14 protein structure verification. [file 7234961.f1.zip › Figure S7_orf9b tunnel1-profile.pptx]

## Slide 1
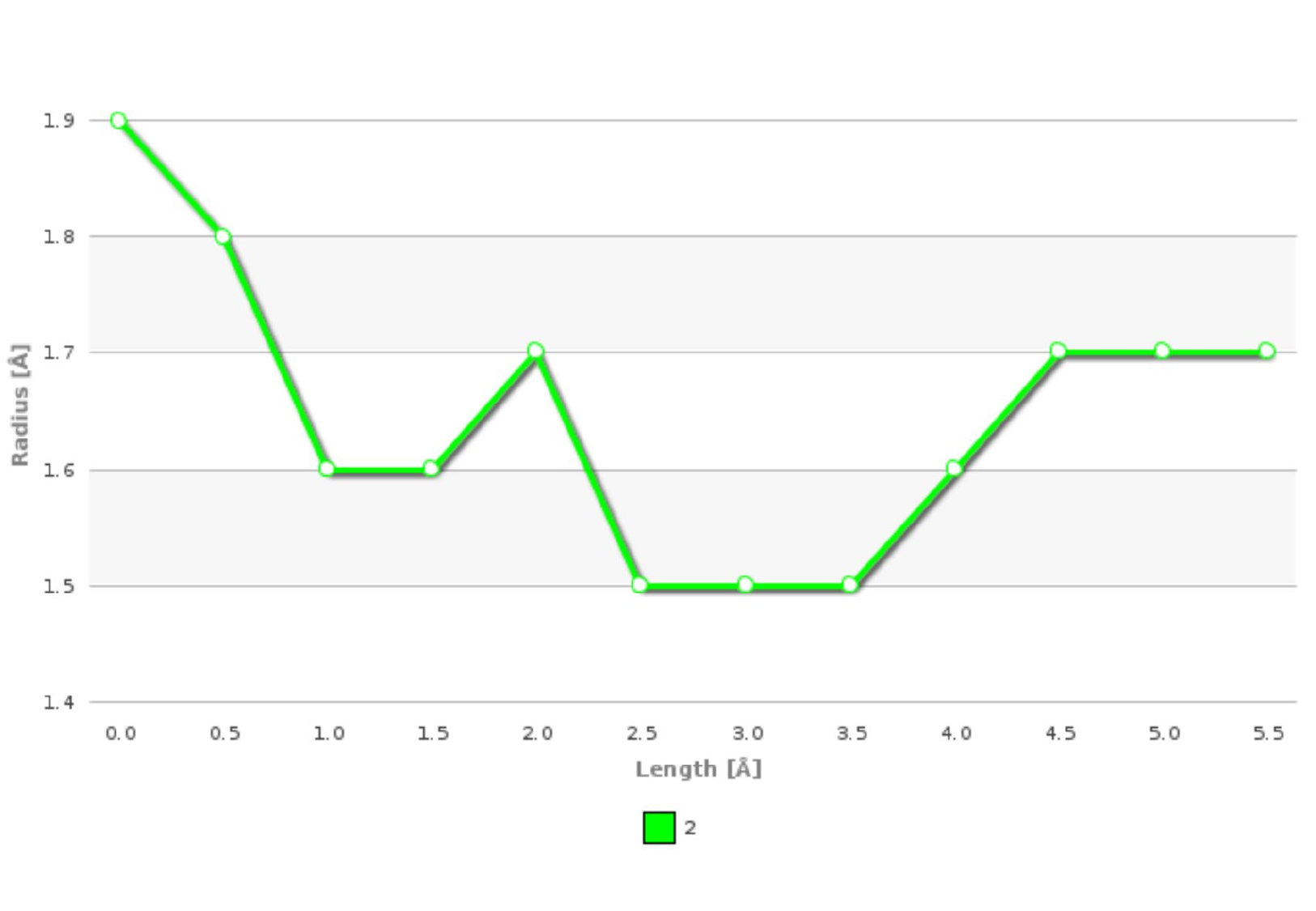

Supplement: Supplementary Materials — Table S1: computed cavities in the 3D structure of ORF9b protein for active sites. Table S2: computed cavities in the 3D structure of ORF14 protein for active sites. Figure S1: secondary structure profile of 9b protein. Figure S2: secondary structure profile of ORF14 protein. Figure S3: QMEANDisCo local quality estimate for 9b protein. Figure S4: QMEANDisCo local quality estimate for ORF14 protein. Figure S5: protein 9b structure verification in ERRAT. Figure S6: protein ORF14 structure verification in ERRAT. Figure S7: profile of tunnel 1 in 9b protein. Figure S8: profile of tunnel 2 in 9b protein. Figure S9: tunnel-profile of ORF14 protein. Figure S10: hydropathicity plot for 9b protein. Figure S11: hydrophobicity plot for ORF14 protein. Annexure 1: protein 9b structure verification. Annexure 2: ORF14 protein structure verification. [file 7234961.f1.zip › Figure S8_orf9b tunnel2-profile.pptx]

## Slide 1
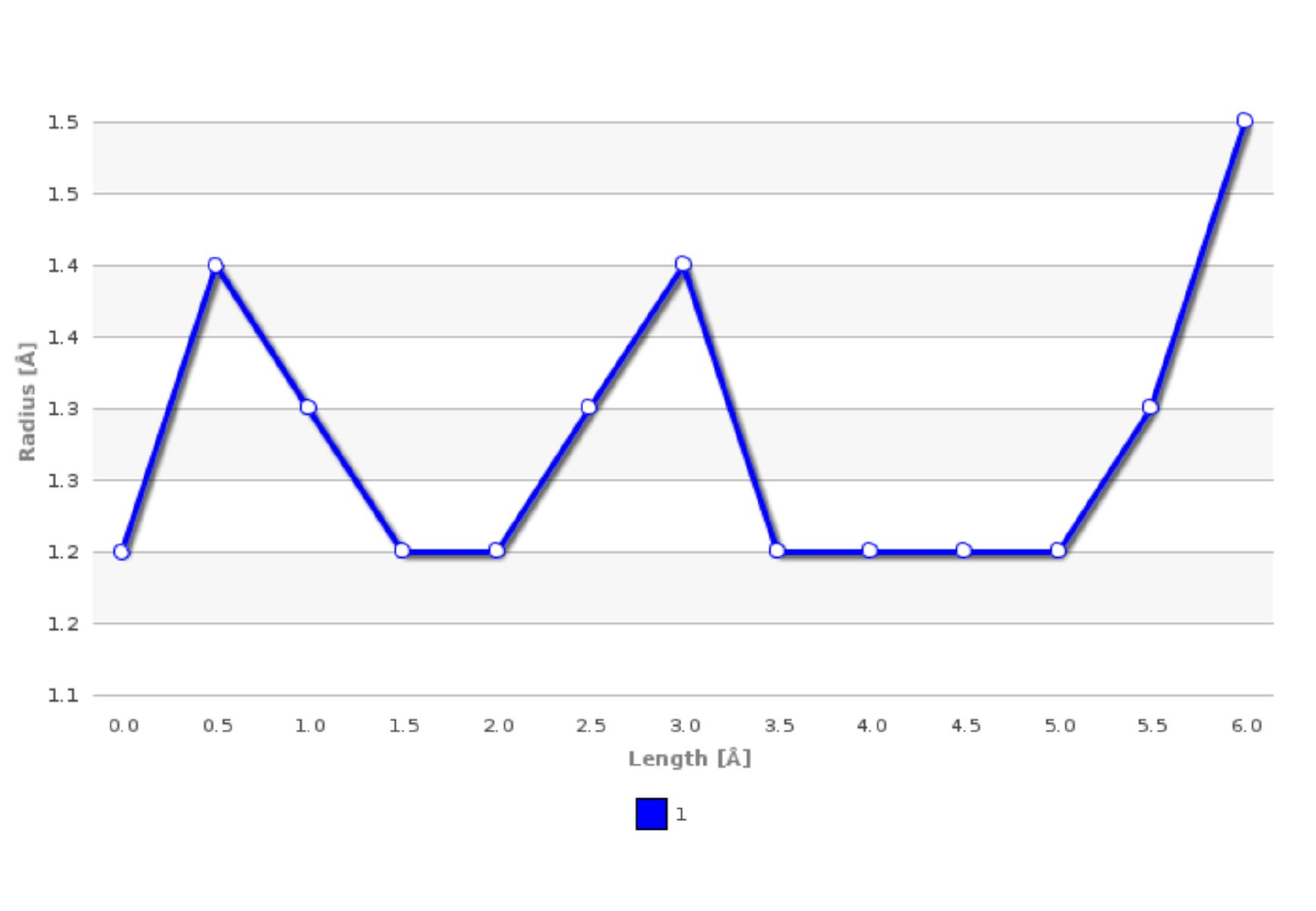

Supplement: Supplementary Materials — Table S1: computed cavities in the 3D structure of ORF9b protein for active sites. Table S2: computed cavities in the 3D structure of ORF14 protein for active sites. Figure S1: secondary structure profile of 9b protein. Figure S2: secondary structure profile of ORF14 protein. Figure S3: QMEANDisCo local quality estimate for 9b protein. Figure S4: QMEANDisCo local quality estimate for ORF14 protein. Figure S5: protein 9b structure verification in ERRAT. Figure S6: protein ORF14 structure verification in ERRAT. Figure S7: profile of tunnel 1 in 9b protein. Figure S8: profile of tunnel 2 in 9b protein. Figure S9: tunnel-profile of ORF14 protein. Figure S10: hydropathicity plot for 9b protein. Figure S11: hydrophobicity plot for ORF14 protein. Annexure 1: protein 9b structure verification. Annexure 2: ORF14 protein structure verification. [file 7234961.f1.zip › Figure S9_orf14 protin tunnel-profile.pptx]
